# Mo_2_TiC_2_ MXene-Supported Ru Clusters for Efficient Photothermal Reverse Water–Gas Shift

**DOI:** 10.1021/acsnano.2c10707

**Published:** 2022-12-30

**Authors:** Zhiyi Wu, Jiahui Shen, Chaoran Li, Chengcheng Zhang, Kai Feng, Zhiqiang Wang, Xuchun Wang, Debora Motta Meira, Mujin Cai, Dake Zhang, Shenghua Wang, Mingyu Chu, Jinxing Chen, Yuyao Xi, Liang Zhang, Tsun-Kong Sham, Alexander Genest, Günther Rupprechter, Xiaohong Zhang, Le He

**Affiliations:** † Institute of Functional Nano & Soft Materials (FUNSOM), Soochow University-Western University Centre for Synchrotron Radiation Research, Soochow University, Suzhou 215123, PR China; ‡ Jiangsu Key Laboratory of Advanced Negative Carbon Technologies, Soochow University, Suzhou, 215123 Jiangsu. PR China; § Department of Chemistry, Soochow University-Western University Centre for Synchrotron Radiation Research, University of Western Ontario, London, Ontario N6A 5B7, Canada; ∥ CLS@APS, Advanced Photon Source, Argonne National Laboratory, Lemont, Illinois 60439, United States; ⊥ Institute of Materials Chemistry, Technische Universität Wein, Wien 1060, Austria

**Keywords:** Photothermal catalysis, MXene materials, supported metal clusters, CO_2_ hydrogenation, solar fuels

## Abstract

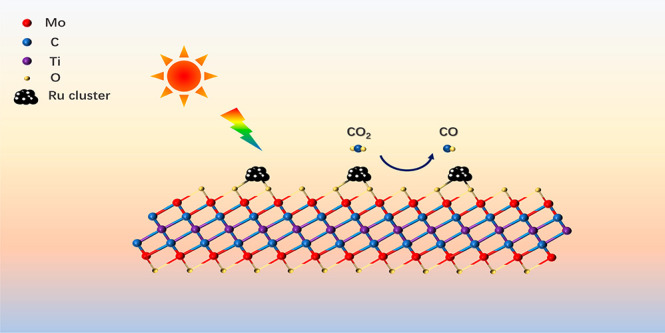

Driving metal-cluster-catalyzed high-temperature chemical reactions by sunlight holds promise for the development of negative-carbon-footprint industrial catalysis, which has yet often been hindered by the poor ability of metal clusters to harvest and utilize the full spectrum of solar energy. Here, we report the preparation of Mo_2_TiC_2_ MXene-supported Ru clusters (Ru/Mo_2_TiC_2_) with pronounced broadband sunlight absorption ability and high sintering resistance. Under illumination of focused sunlight, Ru/Mo_2_TiC_2_ can catalyze the reverse water–gas shift (RWGS) reaction to produce carbon monoxide from the greenhouse gas carbon dioxide and renewable hydrogen with enhanced activity, selectivity, and stability compared to their nanoparticle counterparts. Notably, the CO production rate of MXene-supported Ru clusters reached 4.0 mol·g_Ru_^–1^·h^–1^, which is among the best reported so far for photothermal RWGS catalysts. Detailed studies suggest that the production of methane is kinetically inhibited by the rapid desorption of CO from the surface of the Ru clusters.

## Introduction

Industrial heterogeneous catalysis plays a vital role in the manufacturing of fuels and commodity, specialty, and fine chemicals.^1−3^ Many industrial catalytic processes require operation under harsh conditions such as high temperatures and pressures, resulting in huge fossil energy consumption and carbon footprints.^4−6^ For example, ammonia production alone is responsible for over 1% of the global energy consumption and annual CO_2_ emission of 420 million tons.^7,8^ There is an urgent need for alternative catalysts with improved activity and selectivity to reduce the energy consumption associated with industrial catalysis. Moreover, light-assisted catalysis has attracted tremendous attention since it allows for partial or even entire replacement of fossil energy consumption with solar irradiation.^9−17^ For example, the transient or permanent transfer of photoexcited charge carriers from the catalysts to adsorbed reactants, that is, photochemical activation, can significantly lower the energy barrier of a high-temperature reaction and allow operation under milder conditions.^18−21^ Sunlight-driven catalysis can also make use of the local photothermal effect of metal nanoparticles. This photothermal approach harvests both light and heat from solar irradiation, enabling more efficient utilization of the whole solar spectrum.^22−27^ Thanks to the localized heating of catalysts by sunlight, photothermal catalysis could in principle operate at ambient temperatures and conserve energy by avoiding the need to heat the reactor, which is necessary for thermocatalysis.^8,23,28−31^ Ideally, negative-carbon-footprint industrial catalysis should feature the use of more efficient catalysts while being fully driven by solar energy. Such transition from fossil-driven high-temperature thermocatalysis to sunlight-driven photothermal catalysis is of great significance toward carbon neutrality in the near future.

In the quest for alternatives to industrial metal nanocatalysts, supported metal clusters (SMCs) with reduced sizes (<2 nm) have recently emerged.^32−34^ Owing to the abundance of adjacent metal–metal bonds, as well as great geometrical and electronic structures, SMCs have exhibited enhanced catalytic activity and selectivity in a wide range of reactions.^35−39^ For example, it was reported that α-MoC supported Pt clusters (Pt/α-MoC) could catalyze the water–gas shift (WGS) reaction at a low temperature of 40 °C through a hydrogen-production pathway involving direct CO dissociation.^40^ Intuitively, the combination of SMCs with sunlight-driven catalysis would allow for a further reduction of energy consumption. Nevertheless, compared with nanoparticle counterparts, SMCs suffer from much lower metal loading and poorer sunlight absorption ability owing to their weaker localized surface plasmon resonance (LSPR) effect and limited absorption cross-section.^25^ Therefore, it is highly desired but challenging to develop efficient photothermal catalysts based on SMCs that can effectively harvest and utilize the full spectrum of solar energy.

Here we propose a concept of efficient photothermal catalysis over metal clusters supported by carbide MXenes. The strong metal–support binding may enable a high dispersion of metals on the MXene surface.^41^ Meanwhile, the use of MXene supports with excellent photothermal properties can significantly improve sunlight absorption ability and photothermal conversion efficiency of SMCs catalysts.^42−45^ As a proof-of-concept, we demonstrate the preparation of Mo_2_TiC_2_ MXene supported Ru clusters (Ru/Mo_2_TiC_2_) with a relatively high metal loading of 2.1 wt % and strong broadband sunlight absorption. X-ray photoelectron and absorption spectroscopy studies reveal the existence of a possible Ru/RuO_*x*_/MoO_*x*_/Mo_2_TiC_2_ interface in Ru/Mo_2_TiC_2_, responsible for its strong metal–support binding and sintering resistance even at 500 °C. Powered solely by sunlight, Ru/Mo_2_TiC_2_ can efficiently, selectively, and robustly catalyze the reverse WGS reaction under ambient conditions, which is different from Ru photothermal catalysts in the literature favoring the methanation reaction.^46−48^ Detailed studies suggest that the production of methane is kinetically inhibited by the rapid desorption of CO from the surface of the Ru clusters.

## Results and Discussion

**Figure 1:**
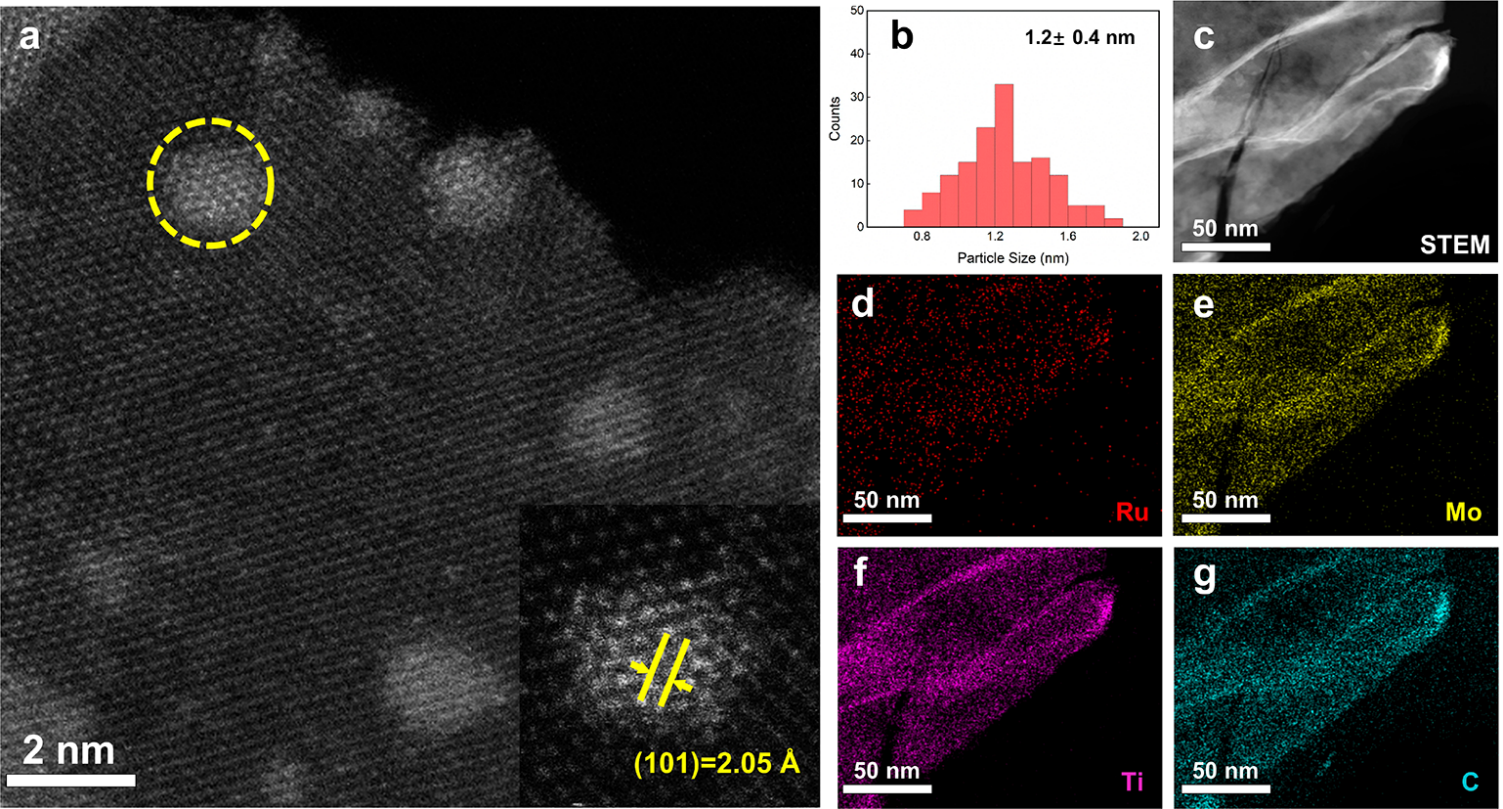
Characterization of Ru/Mo_2_TiC_2_. (a) HAADF-STEM image of Ru/Mo_2_TiC_2_ (inset shows the lattice spacing of (101) planes of metallic Ru). (b) Size histogram of Ru clusters. (c–f) Elemental mapping of Ru/Mo_2_TiC_2_.

Among various MXene materials, Mo_2_TiC_2_ was chosen as support for Ru clusters in this study due to its relatively high stability against oxidization. Specifically, few-layered two-dimensional Mo_2_TiC_2_ nanosheets were obtained via a modified chemical exfoliation method (Figures S1–S3).^49−51^ Ru clusters were then loaded onto Mo_2_TiC_2_ through adsorption of Ru^3+^, followed by the subsequent reduction by H_2_ at 500 °C (Figure S4). Transmission electron microscopy (TEM) images of the as-obtained sample, henceforth denoted as Ru/Mo_2_TiC_2_, revealed the successful loading of ultrasmall Ru nanocrystals on Mo_2_TiC_2_ nanosheets with the morphology and crystalline structure of the MXene support remaining intact (Figure S5). High-angle annular dark-field scanning transmission electron microscopy (HAADF-STEM) further confirmed the formation of highly dispersed Ru clusters on Mo_2_TiC_2_ nanosheets (Figure 1a). The distance between two adjacent planes was found to be 0.205 nm, which is in accordance with the lattice spacing of the (101) plane of metallic Ru (Figure 1a, inset). The average size of Ru, obtained from analysis of 150 particles was found to be 1.2 ± 0.4 nm, a size in the cluster range (Figure 1b, Figure S5a).^52^ The X-ray diffraction (XRD) pattern of Ru/Mo_2_TiC_2_ did not show peaks characteristic of metallic Ru, which is consistent with a metal loading of 2.1 wt % determined by inductively coupled plasma-mass spectrometry (Figure S6).^53^ Energy-dispersive X-ray (EDX) elemental mapping further evidenced a uniform distribution of Ru clusters on the support (Figure 1c–g). Notably, Ru/Mo_2_TiC_2_ was prepared at a relatively high temperature of 500 °C, confirming its high thermal stability against sintering.

A strong metal–support binding between Ru and Mo_2_TiC_2_ MXene is key for the preparation of highly dispersed and sintering-resistant Ru clusters. For comparison, an impregnated Ru/silica catalyst with a similar metal loading (2 wt %), denoted as Ru-NP/SiO_2_-1, was prepared under the same reduction temperature of 500 °C (Table S1). The size of the Ru particles in Ru-NP/SiO_2_-1 was found to be 4.3 ± 0.6 nm, far above the size range of clusters on Mo_2_TiC_2_ MXene (Figure S7, Table S1). Smaller Ru particles (3.2 ± 0.6 nm) were obtained, denoted as Ru-NP/SiO_2_-2, under the same conditions except with a lowered Ru loading to 0.5 wt % (Figure S8). In our experiments, H_2_ reduction at a temperature as low as 200 °C was required to produce Ru clusters, denoted as Ru/SiO_2_, with an average size of 1.7 ± 0.3 nm (Figure S9). In sharp contrast to the difficulty of preparing silica supported Ru clusters, with the Mo_2_TiC_2_ support the size of Ru particles only increased to 2.4 nm even for a Ru loading up to 7.8 wt % (Figure 1b, Figures S10–S11). This 7.8 wt % sample is denoted as Ru-NP/Mo_2_TiC_2_. CO temperature-programmed desorption (CO-TPD) was also used to compare the Ru dispersity and thus the Ru particle size in Ru/Mo_2_TiC_2_ and Ru-NP/Mo_2_TiC_2_ (Figure S12 and Table S2). Average Ru nanoparticle sizes calculated from CO chemisorption data and TEM images are compared in Table S2.^54^ Results derived from both techniques are in good agreement and further reveal the high dispersity of Ru nanoclusters supported on Mo_2_TiC_2_ MXenes.

**Figure 2:**
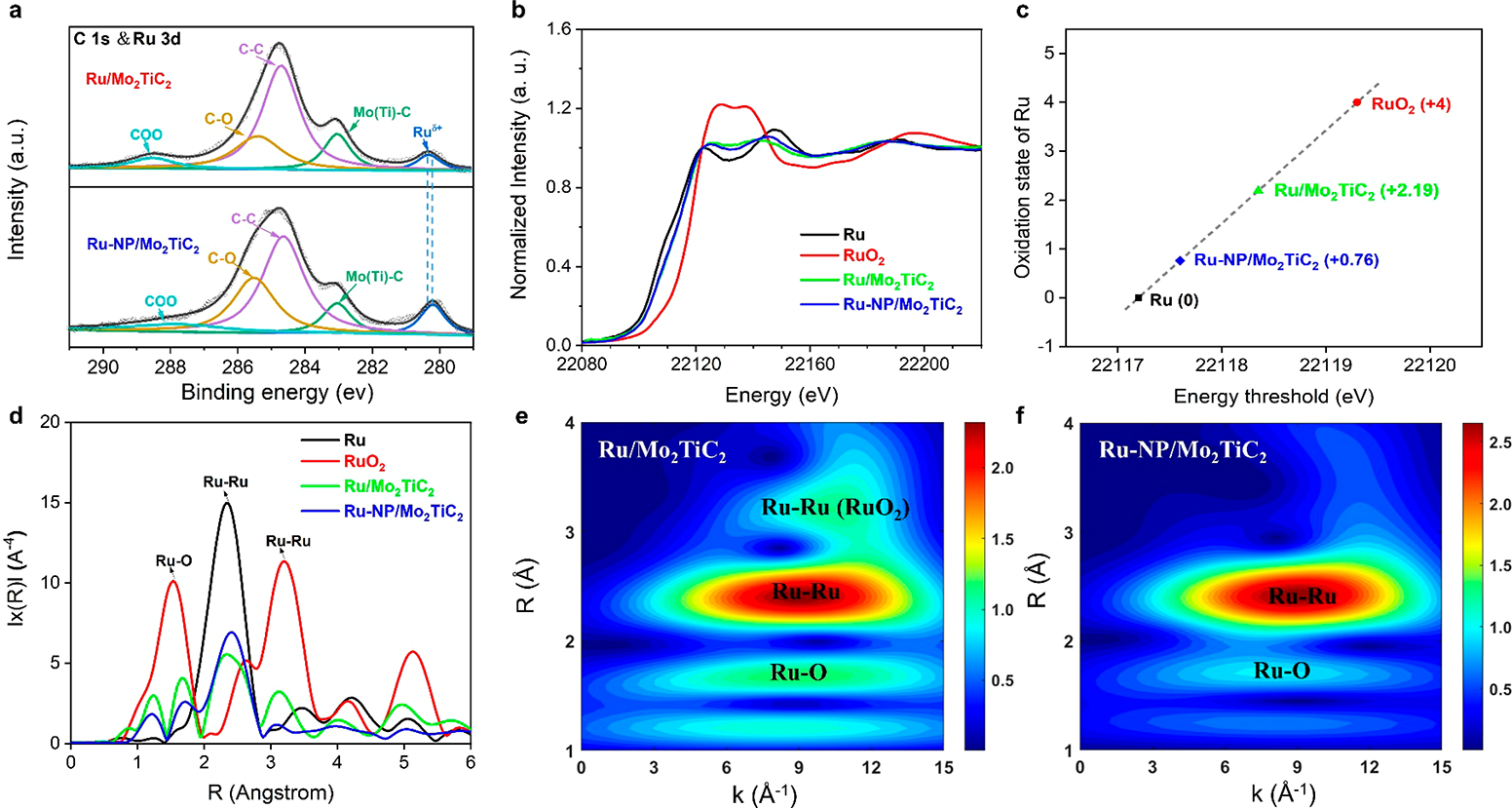
(a) C 1s and Ru 3d core-level XPS spectra of Ru/Mo_2_TiC_2_ and Ru-NP/Mo_2_TiC_2_. Ru/Mo_2_TiC_2_ exhibited a higher Ru oxidation state than Ru-NP/Mo_2_TiC_2_, as indicated by the higher binding energy of the former. (b) Normalized XANES spectra at the Ru K-edge of Ru, RuO_2_, Ru/Mo_2_TiC_2_, and Ru-NP/Mo_2_TiC_2_. (c) Oxidation states of Ru for reference and synthesized materials as determined from the edge positions in the Ru K-edge XANES spectra. (d) Corresponding FT-EXAFS spectra derived from the Ru K-edge of Ru, RuO_2_, Ru/Mo_2_TiC_2_ and Ru-NP/Mo_2_TiC_2_. (e, f) Wavelet transformation for the Ru K-edge EXAFS signals of Ru/Mo_2_TiC_2_ and Ru-NP/Mo_2_TiC_2_, respectively, further confirming a higher Ru oxidation state in Ru/Mo_2_TiC_2_.

X-ray photoelectron spectroscopy (XPS), X-ray absorption near-edge structure (XANES), and extended X-ray absorption fine structure (EXAFS) were collectively used to study the interface between Ru clusters and Mo_2_TiC_2_ MXene. It was found that Ru species were partially oxidized with an average oxidation state higher than 0 but much lower than +4 for both Ru/Mo_2_TiC_2_ and Ru-NP/Mo_2_TiC_2_ (Figure 2a–d, Figures S13–S15).^55^ This is consistent with a previous report showing the existence of strong electronic interaction between Ru nanoparticles and few-layer Mo_2_TiC_2_ MXenes.^51^ As shown in Figure 2b, Ru K-edge XANES of both Ru/Mo_2_TiC_2_ and Ru-NP/Mo_2_TiC_2_ shows similar features to that of metallic Ru, suggesting that the majority of Ru in both samples exists as Ru(0). On closer look, the energy threshold of Ru/Mo_2_TiC_2_ and Ru-NP/Mo_2_TiC_2_ slightly shifts to higher energy, and the two main features become broadened and closer to each other compared to metallic Ru, which indicates that the Ru in both samples was partially oxidized. Based on the energy threshold position that increases with oxidation state (Figure 2c), Ru/Mo_2_TiC_2_ exhibited a higher Ru oxidation state than Ru-NP/Mo_2_TiC_2_ (+2.19 vs +0.76). The R space and wavelet transformation of Ru K-edge EXAFS (Figure 2d–f) also show that Ru/Mo_2_TiC_2_ has stronger Ru–O scattering than Ru-NP/Mo_2_TiC_2_, which agrees well with the XANES analysis. Moreover, the oxidation state of Mo also increased after loading Ru onto Mo_2_TiC_2_ MXene, as revealed by Mo 3d XPS and Mo K-edge XANES (Figures S16–S21). Different interfaces between metal and carbide MXenes were reported in previous studies, including the formation of Pd–Nb alloy in Nb_2_CT_*x*_ supported Pd nanoparticle and AuC_*x*_ (metal carbides) in MoC_*x*_ supported Au nanoparticles.^41,56^ The formation of RuMo alloy or RuC_*x*_ seems contradictory with our XPS and XANES results. While further studies are needed to elucidate the interfacial structure, we infer that a plausible Ru/RuO_*x*_/MoO_*x*_/Mo_2_TiC_2_ interface might be formed in our Mo_2_TiC_2_ supported Ru samples. Compared with Ru-NP/Mo_2_TiC_2_, Ru/Mo_2_TiC_2_ exhibited a higher proportion of interfacial and surficial Ru atoms, which is responsible for the higher oxidation state of Ru (Figures S18–S21).

**Figure 3:**
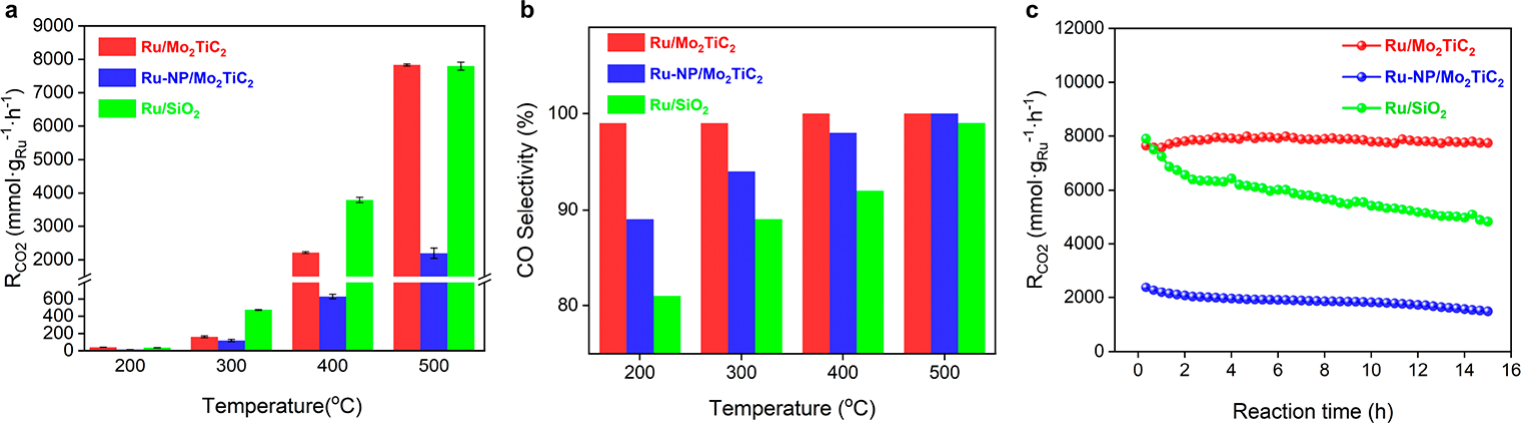
(a, b) Thermocatalytic activity and CO selectivity of Ru/Mo_2_TiC_2_, Ru-NP/Mo_2_TiC_2_, and Ru/SiO_2_ catalysts at different temperatures. (c) Thermocatalytic performance of Ru/Mo_2_TiC_2_ in catalyzing CO_2_ hydrogenation at 500 °C under dark conditions for a continuous 15 h flow reactor test.

Thermocatalytic reverse water–gas shift (RWGS) was chosen as a model reaction to demonstrate the intrinsically high activity, selectivity, and stability of MXene supported Ru clusters (Figure S22). CO and CH_4_ were the only detected products for all samples. Pure Mo_2_TiC_2_ nanosheets are proven to be almost inert for CO_2_ hydrogenation reactions (Figures S23 and S24). In the investigated temperature range of 200–500 °C, Ru/Mo_2_TiC_2_ exhibited a higher CO_2_ conversion rate (normalized by metal loading) and conversion degree than Ru-NP/Mo_2_TiC_2_ and also near-unity CO selectivity (Figure 3a,b and Table S3). Although Ru/SiO_2_ exhibited the highest activity below 500 °C among the three catalysts, its CO selectivity was slightly lower than that of Ru/Mo_2_TiC_2_. We performed CO_2_ temperature-programmed desorption (TPD) experiments over Ru/Mo_2_TiC_2_, Ru-NP/Mo_2_TiC_2_ and Ru/SiO_2_ catalysts.^57^ Both MXene-supported samples exhibited enhanced adsorption and activation of CO_2_, as revealed by larger peak areas and higher complete desorption temperatures (Figure S25). It is more likely that the active sites for CO_2_ hydrogenation locate at the Ru surface and the catalytic activity is mainly determined by the number of active sites and the hydrogenation ability of different Ru catalysts. Owing to the weak metal–support interaction, Ru/SiO_2_ exhibited a more metallic state of Ru, facilitating hydrogen dissociation. The enhanced hydrogenation ability of Ru/SiO_2_, as revealed by H_2_-TPD results (Figure S26), improves the catalytic activity and favors the deep hydrogenation of CO_2_ to produce methane (Figure 3b). With Ru/Mo_2_TiC_2_ as catalyst, apart from the increased number of active sites, the apparent activation energy of RWGS was also found to be lower than that of Ru-NP/Mo_2_TiC_2_ (Figure S27). Moreover, the catalytic performance of Ru/Mo_2_TiC_2_ remained stable in a continuous 15 h run at 500 °C, while a gradual deactivation was observed for both Ru-NP/Mo_2_TiC_2_ and Ru/SiO_2_ (Figure 3c). TEM studies revealed that the size of ruthenium particles in Ru/Mo_2_TiC_2_ remained below 2 nm throughout the stability test (Figure S28). In contrast, an obvious size increase in Ru particles was observed for both spent Ru-NP/Mo_2_TiC_2_ and Ru/SiO_2_ catalysts (Figures S29 and S30).

While ruthenium nanoparticles are known to be favorable for deep hydrogenation of CO_2_ to methane due to their strong hydrogenation ability, Ru clusters with reduced size may selectively produce CO.^22,46,58^ It is found that further CO hydrogenation to CH_4_ by Ru/Mo_2_TiC_2_ is kinetically inhibited by the rapid desorption of CO from the partially oxidized Ru surface despite its strong CO dissociation ability. Our previous study suggested that the ability of H_2_-assisted CO dissociation by Ni/MgAl_2_O_4_ catalysts, which strongly depends on the size and oxidation state of Ni nanoparticles, controls the production of CH_4_.^59^ In the current study, an opposite trend was observed in CO hydrogenation temperature-programmed surface reaction (TPSR) experiments (Figure S31). MXene supported Ru clusters with a higher oxidation state of Ru exhibited a slightly lower activation temperature and thus stronger ability for H_2_-assisted CO dissociation than Ru nanoparticles. CO temperature-programmed desorption (TPD) experiments suggested that CO desorption from Ru/Mo_2_TiC_2_ occurs at a lower temperature than from Ru-NP/Mo_2_TiC_2_. This observation is consistent with weaker binding of CO to partially oxidized Ru surfaces (Figure S12). It is most likely that the product selectivity of MXene supported Ru catalysts is controlled by the competition between desorption and dissociation of CO.

**Figure 4:**
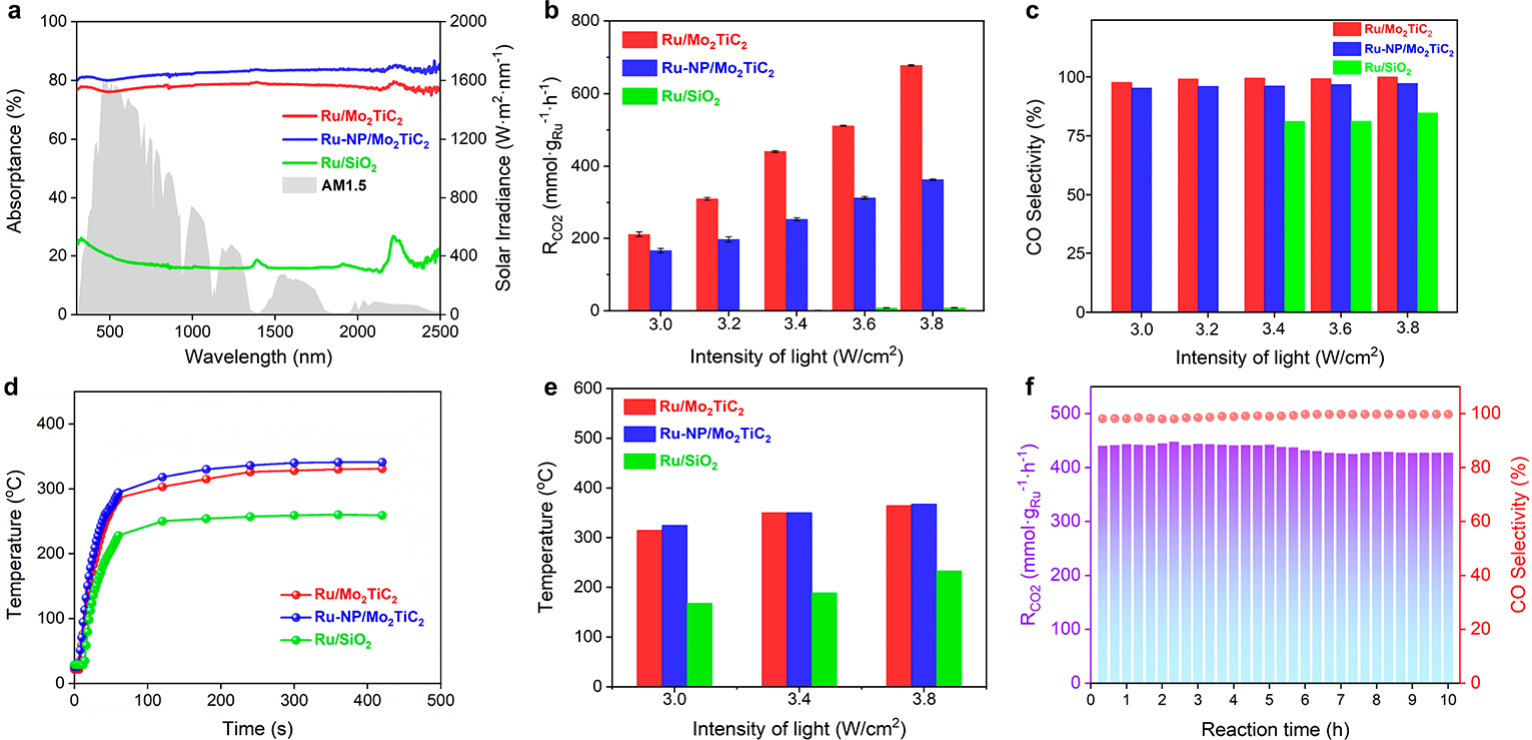
(a) Diffuse reflectance spectra of Ru/Mo_2_TiC_2_, Ru-NP/Mo_2_TiC_2_, and Ru/SiO_2_, and the spectrum of AM 1.5G. (b, c) Photothermal catalytic activity and CO selectivity of Ru/Mo_2_TiC_2_, Ru-NP/Mo_2_TiC_2_, and Ru/SiO_2_ under different illumination conditions. The concentration of both CO and CH_4_ products of Ru/SiO_2_ were below the detection limit of the gas chromatography under low light intensities (<3.2 W/cm^2^) so that the CO selectivity could not be determined. (d) Surface temperature profiles (*T*_sur_) of Ru/Mo_2_TiC_2_, Ru-NP/Mo_2_TiC_2_, and Ru/SiO_2_ catalysts under the illumination of 3.0 W/cm^2^. (e) Equivalent working temperatures (*T*_e_) of Ru/Mo_2_TiC_2_, Ru-NP/Mo_2_TiC_2_, and Ru/SiO_2_ catalysts under different light intensities. (f) Photothermal catalytic performance of Ru/Mo_2_TiC_2_ under 3.4 W/cm^2^ illumination for continuous 10 h testing in the flow reactor. Testing conditions: the flow rates of H_2_, CO_2_, and N_2_ were 5, 5, and 10 mL/min, respectively; no external heating was provided.

The use of Mo_2_TiC_2_ MXene as a support not only enables the preparation of highly dispersed and thermally stable Ru clusters but also significantly improves the sunlight absorption ability. Notably, MXenes are well-known excellent photothermal materials with strong electromagnetic wave absorption capacity.^43,60−62^ Compared with Ru/SiO_2_ of almost white color, the black Ru/Mo_2_TiC_2_ reached nearly 80% absorption efficiency across the whole solar spectrum (Figure 4a). Owing to its higher Ru loading, Ru-NP/Mo_2_TiC_2_ exhibited slightly stronger sunlight absorption than Ru/Mo_2_TiC_2_, further corroborating the advantage of MXene materials over conventional oxide supports.

Compared with conventional SMCs, the significantly enhanced sunlight harvesting ability and photothermal conversion efficiency in Ru/Mo_2_TiC_2_ lays the foundation for the development of efficient sunlight-driven metal cluster catalysis. In the absence of external heating, under the same light intensities Ru/Mo_2_TiC_2_ catalyzed photothermal CO_2_ hydrogenation to produce CO at much higher rates, conversion degree, and selectivity than Ru/SiO_2_, despite their similar intrinsic catalytic reactivity. (Figure 4b,c and Table S4). Notably, the CO_2_ rate, *R*_CO_2__, was found to be as high as 678 mmol·g_Ru_^–1^·h^–1^ for Ru/Mo_2_TiC_2_ under 3.8 W/cm^2^ illumination in the flow reactor, 2 orders of magnitude increase over Ru/SiO_2_. The use of carbon-13 labeled carbon dioxide (^13^CO_2_) as an isotope tracer molecule confirmed that both CO and CH_4_ products originated from CO_2_ (Figure S32). Both the surface temperature (*T*_sur_) measured by a thermocouple and the equivalent working temperature (*T*_e_) of the illuminated Ru/Mo_2_TiC_2_ catalyst were over 100 °C higher than that of silica supported Ru clusters (Figure 4d,e). It is also important to note that the Ru/Mo_2_TiC_2_ catalyst exhibited a near-unity CO selectivity under all investigated light intensities, which is different from previously reported Ru photothermal catalysts favoring the production of methane (Table S4). More CH_4_ production was observed for Ru/SiO_2_, which could mainly be attributed to the formation of larger Ru particles under testing conditions (Figure S33).

MXene supported Ru clusters also exhibited better photothermal catalytic performance, in terms of CO production rate and selectivity, than their nanoparticle counterparts with a higher Ru loading under the same conditions (Figure 4b,c). As discussed above, the Ru/Mo_2_TiC_2_ catalyst exhibited enhanced intrinsic activity and selectivity but slightly weaker sunlight absorption ability than Ru-NP/Mo_2_TiC_2_. It was found that Ru-NP/Mo_2_TiC_2_ reached slightly higher *T*_sur_ and *T*_e_ than Ru/Mo_2_TiC_2_ under the same illumination (Figure 4d,e). However, this advantage in the local temperature of Ru catalysts seemed less important than the intrinsic reactivity in determining the overall photothermal catalytic activity. In other words, both the excellent photothermal properties and intrinsically high catalytic reactivity of Ru/Mo_2_TiC_2_ are vital for efficient sunlight-driven metal cluster catalysis.

Since the desorption of CO is kinetically more favored than its further hydrogenation, Ru/Mo_2_TiC_2_ exhibited near-unity CO selectivity in both thermocatalytic and photothermal catalytic RWGS reactions at low CO_2_ conversions (<0.5% for the flow reactor). Upon increasing CO_2_ conversion, the CO concentration in the reactor apparently increases, increasing the possibility of further CO hydrogenation to CH_4_. The photothermal RWGS reaction catalyzed by Ru/Mo_2_TiC_2_ was also carried out in a batch reactor (Figure S34). Under 3.8 W/cm^2^ illumination of simulated sunlight for 5 min, the CO_2_ conversion increased to 2.0%, while the CO selectivity dropped to 90% with CH_4_ as the only detectable byproduct. Notably, the CO production rate reached 4.0 mol·g_Ru_^–1^·h^–1^ in the batch reactor, which is among the best reported so far for photothermal RWGS catalysts (Table S5).

The strong metal–support binding between Ru and Mo_2_TiC_2_ MXene ensures the long-term durability of Ru clusters at high local temperatures. In addition to its enhanced activity and selectivity, Ru/Mo_2_TiC_2_ also exhibited high stability in photothermal catalysis. No obvious decrease in either CO production rate or selectivity was observed for continuous 10 h testing of photothermal catalysis under a light intensity of 3.4 W/cm^2^ (Figure 4f). TEM studies revealed that the morphology and structure of spent Ru/Mo_2_TiC_2_ remained intact (Figure S35). After the cycling test, the average size of Ru particles slightly increased to 1.7 nm, still falling into the size range of clusters. In contrast, both the activity and selectivity dropped for Ru-NP/Mo_2_TiC_2_, accompanied by the increase of Ru size from 2.4 to 3.0 nm, under the same conditions (Figures S36 and S37). This observation is consistent with the results of thermocatalytic testing, further confirming the stronger binding of Ru clusters with the MXene support.

It appeared in our study that photons in long wavelengths made a major contribution to the sunlight-driven RWGS catalysis over Mo_2_TiC_2_ MXene supported Ru clusters. Control experiments were carried out to compare the effects of exposing ultraviolet–visible (UV–vis, λ < 800 nm) or infrared (IR, λ > 800 nm) light to the Ru/Mo_2_TiC_2_ catalyst under conditions of external heating. In the temperature range of 200–300 °C, the catalytic activity improved after applying either UV–vis or IR light with the same intensity of 0.36 W/cm^2^, but the enhancement was more pronounced for the latter (Figure S38a,b). Specifically, an increase of ∼10% in activity was observed for the UV–vis light. In contrast, the enhancement factor of the IR light, defined as the ratio of light activity to dark activity, increased monotonically from ∼1.1 at 200 °C to ∼1.6 at 300 °C. We also introduced monochromatic light sources (365 nm, 520 nm, 620 nm and 850 nm) with the same intensity of 0.36 W/cm^2^ during the thermocatalytic reaction (Figure S38c,d). No obvious change in either the activity or selectivity was observed under the 365 nm illumination, suggesting a very minor contribution of UV light. As the wavelength increased, the light-induced enhancement of catalytic activity was more pronounced. Apparently, the thermal effect of light is dominant in the photothermal RWGS reaction over Ru/Mo_2_TiC_2_ although a nonthermal effect cannot be entirely excluded.^63−65^

**Figure 5:**
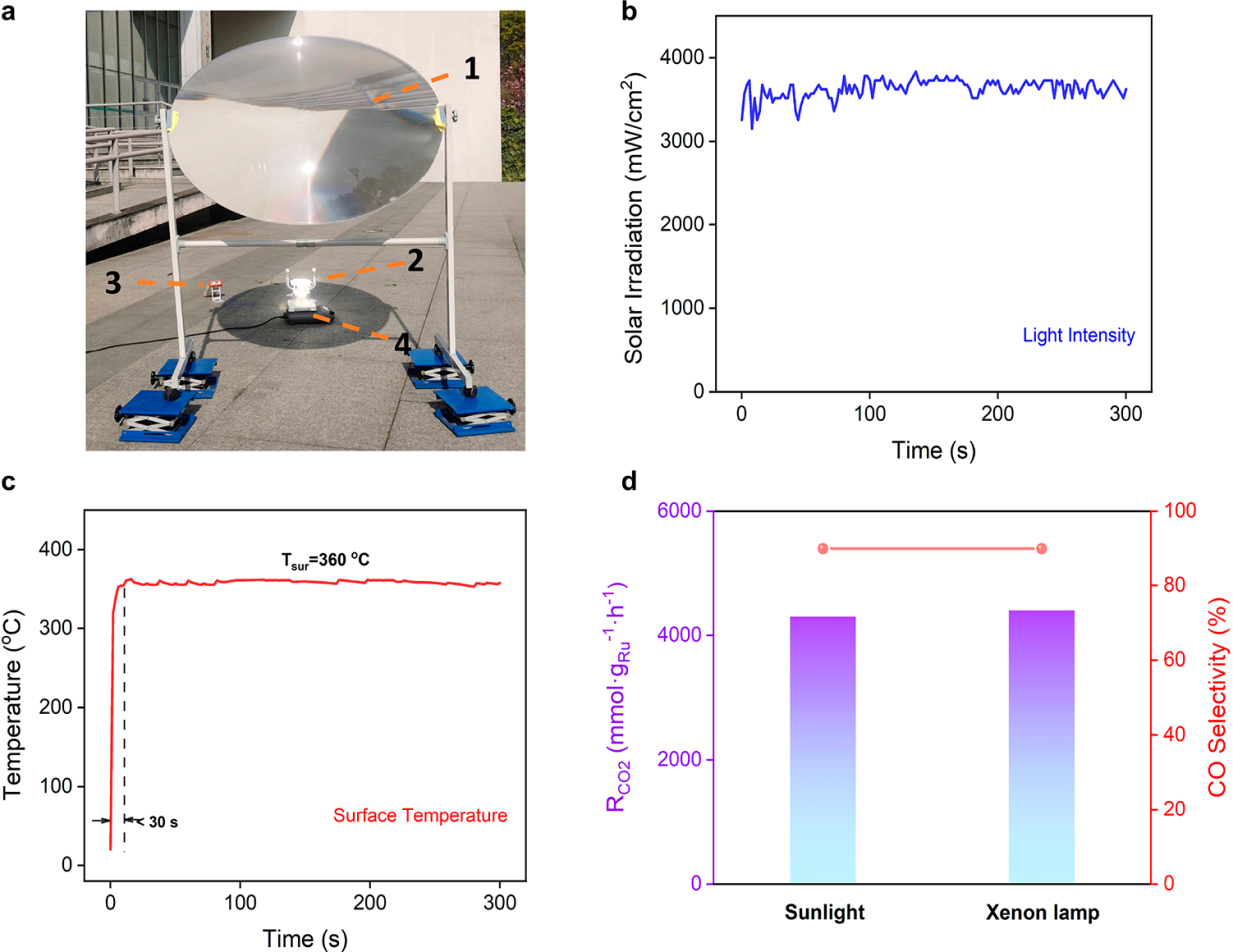
(a) Setup of the outdoor experiments: (1) Fresnel lens (the lens was fabricated on a poly(methyl methacrylate) plate with a diameter of 110 cm), (2) reactor, (3) directly contacting thermocouple equipped with a temperature sensor used to probe the surface temperature of the catalyst under the condition of reactions,^66,67^ (4) magnetic stirrer to enhance gas diffusion in the reactor. (b) Solar irradiation of the reaction system augmented by the Fresnel lens. (c) Surface temperature profile of the outdoor experiment. (d) CO_2_ conversion and product selectivity of Ru/Mo_2_TiC_2_ under different illumination conditions. Natural sunlight: the experiment started at 15:27 on April 2, 2022, in Suzhou. The solar flux reached an average value of 70 mW/cm^2^. Simulated sunlight: the experiment was tested under illumination of 3.8 W/cm^2^ driven by the 300 W xenon lamp in a batch reactor.

Finally, we demonstrated photothermal CO_2_ catalysis over MXene-supported Ru clusters under natural sunlight. An outdoor experiment was performed in a sealed batch reactor containing the Ru/Mo_2_TiC_2_ catalyst, CO_2_, and H_2_ (Figure S39).^66^ A Fresnel lens was used as optical concentrator to increase the intensity of sunlight illuminating the catalyst surface at 3.7 W/cm^2^ (Figure 5a,b). The surface temperature of Ru/Mo_2_TiC_2_ measured by a thermocouple quickly increased to ∼360 °C within 30 s, which is indicative of the excellent photothermal conversion property of MXene supported clusters (Figure 5c). After 5 min of illumination, both CO and CH_4_ were detected as products. The CO production rate reached 3.9 mol·g_Ru_^–1^·h^–1^ with a CO selectivity of 90%, which almost reproduces the indoor performance under illumination with simulated sunlight of similar intensity (Figure 5d). In other words, the issue of solar intermittency in photothermal catalysis can be addressed by using simulated sunlight during the night or in bad weather. These results not only demonstrate the potential of efficient metal cluster catalysis driven solely by concentrated natural sunlight but also promote continuous and sustainable production of solar fuels.

## Conclusions

In summary, we report efficient sunlight-driven catalysis over MXene supported Ru clusters with increased metal loading, strong light absorption ability, and high sintering resistance. Notably, MXene-supported Ru clusters exhibited excellent photothermal catalytic performance, which is 2.6- and 81-times that of MXene-supported Ru nanoparticles and silica-supported Ru clusters. The key of our strategy lies in the excellent photothermal properties of MXene materials and the strong binding between metal clusters and MXene supports. Notably, the CO production rate of MXene supported Ru clusters reached 4.0 mol·g_Ru_^–1^·h^–1^ in the batch reactor, which is among the best reported so far for photothermal RWGS catalysts. In principle, this strategy can be extended to metal catalysts with even higher dispersion toward the development of efficient sunlight-driven single-atom catalysis. Future studies will focus on the in-depth investigation of the effects of light on the catalytic reactions, aiming at introducing additional photochemical activation to further improve the photocatalytic efficiency. Our study demonstrates the great potential of efficient metal cluster catalysis driven solely by focused natural sunlight. Catalysts that possess improved reactivity and that are exclusively driven by sunlight hold significant promise as a negative-carbon-footprint chemical technology.

## Experimental Section

### Materials

Molybdenum titanium aluminum carbide powders (Mo_2_TiAlC_2_, purity 99%, particle size 200 mesh) were purchased from Beike 2D Materials Co., Ltd. Silicon oxide was purchased from Alfa Aesar Chemicals Co., Ltd. We ground and screened it to obtain silica oxide powders with 80 mesh. Hydrofluoric acid (HF, 49 wt % in water) and tetramethylammonium hydroxide pentahydrate (C_4_H_13_NO·5H_2_O, TMAH, 97%) were purchased from Aladdin, Shanghai Yuanye Bio-Technology Co., Ltd. and Macklin, respectively. Ruthenium(III) trichloride anhydrous (RuCl_3_) was purchased from Aladdin Industrial Corporation. Ethanol and acetone were purchased from Sinopharm Chemical Reagent Co., Ltd. Milli-Q water (Millipore, 18.2 MΩ cm at 25 °C) was used in all experiments.

### Preparation of Mo_2_TiC_2_ MXene (Mo_2_TiC_2_T_*x*_) Nanosheets

Few-layer Mo_2_TiC_2_ nanosheets were synthesized through a modified two-step exfoliation method. First, 0.8 g of Mo_2_TiAlC_2_ powder was mixed with aqueous concentrated HF (49%) in a high-density polyethylene beaker. The mixture was then stirred vigorously at 55 °C for 60 h. The resulting suspension was repeatedly washed with deionized water until the pH of the solution reached ∼7. The obtained suspension was held at −49 °C for 48 h to eliminate the water in the samples. Second, an aqueous solution of TMAH (50 wt %) was added to the freeze-dried sample and stirred for 60 h at room temperature. Subsequently, the products were collected by centrifugation and redispersed in deionized water under an Ar atmosphere for further storage.

### Preparation of Ru/Mo_2_TiC_2_ and Ru-NP/Mo_2_TiC_2_ Catalysts

Anhydrous ruthenium trichloride (20 mg, RuCl_3_) was dispersed in 10 mL of deionized water and sonicated for 30 min, and 2 mL of this solution was added to 100 mg of the obtained few-layer Mo_2_TiC_2_ solution (0.5 mg/mL). The reaction was agitatedly stirred using a Teflon-coated magnet at room temperature for 8 h in a N_2_ atmosphere. The product was precipitated and collected by centrifugation with acetone at 10000 rpm. After drying at 50 °C for 12 h and reduction at 500 °C for 3 h at a heating rate of 2 °C/min in a H_2_ atmosphere, the Mo_2_TiC_2_-based Ru cluster catalyst (Ru/Mo_2_TiC_2_) could be synthesized. Ru-NP/Mo_2_TiC_2_ was obtained by merely varying the addition of aqueous ruthenium chloride to 8 mL. Then, the Mo_2_TiC_2_-based Ru nanoparticle catalyst (Ru-NP/Mo_2_TiC_2_) could also be obtained.

### Preparation of Ru/SiO_2_ Catalysts

The Ru/SiO_2_ catalyst was prepared via a traditional impregnation method using commercial SiO_2_ as support. Specifically, 100 mg of ground silica powder was dissolved in 10 mL of anhydrous ethanol and sonicated for 30 min. Subsequently, different mass fractions of anhydrous ruthenium trichloride were added to the ethanol solution. The dispersion was evaporated at 100 °C under vigorous stirring. The powders were calcinated at 200 °C for 1 h in air at a heating rate of 2 °C/min, followed by calcination at 200 °C for 3 h in H_2_ at a heating rate of 2 °C/min after vacuum-drying at 50 °C for 12 h. Then, the SiO_2_ supported Ru cluster catalyst (Ru/SiO_2_) could be obtained. The SiO_2_-supported Ru particle catalyst (Ru-NP/SiO_2_) was obtained by exposure to a H_2_ atmosphere at 500 °C for 3 h.

## Supporting Information Available

Structural characterization of MXene materials and MXene-based catalysts; configuration of tested catalyst reactors; CO_2_-TPD, CO-TPD, H_2_-TPD, and CO-TPSR profiles of MXene and MXene-based catalysts; photothermal and thermal catalytic testing of MXene and MXene-based catalysts (PDF)
