# Supplementary material for: Mo2TiC2 MXene-Supported Ru Clusters for Efficient Photothermal Reverse Water–Gas Shift
Source: ACS Nano. 2022 Dec 30;17(2):1550–9. doi: 10.1021/acsnano.2c10707 (PMC9878975; doi:10.1021/acsnano.2c10707)
Supplement: Supplementary file 1 — nn2c10707_si_001.pdf [file nn2c10707_si_001.pdf]

Supporting Information

Mo_2TiC_2 MXene-Supported Ru Clusters for Efficient Photothermal Reverse Water Gas Shift

Zhiyi Wu[1,2†], Jiahui Shen[1†], Chaoran Li[1,2], Chengcheng Zhang[1], Kai Feng[1], Zhiqiang Wang[3], Xuchun Wang[3], Debora Motta Meira[4], Mujin Cai[1], Dake Zhang[1], Shenghua Wang[1], Mingyu Chu[1], Jinxing Chen[1], Yuyao Xi[1], Liang Zhang[1,2], Tsun-Kong Sham[3], Alexander Genest[5], Günther Rupprechter[5], Xiaohong Zhang[1,2*] and Le He[1,2*]*

[1]Institute of Functional Nano & Soft Materials (FUNSOM), Soochow University-Western University Centre for Synchrotron Radiation Research, Soochow University, Suzhou, 215123, PR China

[2]Jiangsu Key Laboratory of Advanced Negative Carbon Technologies, Soochow University, Suzhou, 215123, Jiangsu, PR China

[3]Department of Chemistry, Soochow University-Western University Centre for Synchrotron Radiation Research, University of Western Ontario, London, Ontario, Canada, N6A 5B7

[4]CLS@APS, Advanced Photon Source, Argonne National Laboratory, Lemont, IL, 60439, USA

[5]Institute of Materials Chemistry, Technische Universität Wien, Wien 1060, Austria

†These authors contributed equally to this work.

Characterizations

Transmission electron microscopy (TEM) images, EDS mappings and SAED patterns were obtained with an FEI Talos F200X S/TEM at 200 kV with a field-emission gun. Atomic-resolution HAADF-STEM images were obtained using an FEI Titan Cubed Themis G2 S/TEM with a probe corrector and a monochromator, also at 200 kV. X-ray diffraction (XRD) was measured on an Empyrean diffractometer with Cu Kα radiation. Scanning electron microscopy (SEM) images were acquired on a Zeiss Supra 55 instrument (from Carl Zeiss, Germany). XAFS spectroscopy at the K-edge of Mo and Ru was performed on sector 20 BM-B of the Advanced Photon Source (APS; Argonne National Laboratory, USA). The Ru contents of different samples were measured by inductively coupled plasma source mass spectrometry (ICP–MS) (Aurora M90, Jenoptik). The diffuse reflectance spectra of the different samples were recorded using a PerkinElmer Lambda 950 UV/Vis/NIR spectrometer and an integrating sphere of 150 mm diameter. X-ray photoelectron spectroscopy (XPS) data were recorded by a Thermo Scientific ESCALAB 250XI spectrometer equipped with monochromatized Al Kα.

Measurement of X-ray absorption fine structure (XAFS) spectroscopy: XAFS spectroscopy at the K-edge of Mo and Ru was performed on sector 20 BM-B of APS. All samples were ground lightly using a mortar and pestle. The powder was dispersed onto Kapton tape, forming a uniform and homogeneous layer, which was then folded a few times to achieve absorption of at least 20%. Mo K-edge XAFS spectra of Ru/Mo_2TiC_2 and $Ru\text{-}NP/Mo_2TiC_2$ samples as well as standards such as Mo (99.5%, powder, Macklin), MoO_2 (99.9, Alfa Aesar Chemicals Co., Ltd) and MoO_3 (99.9% metal basis, Macklin) were recorded in transmission mode by measuring the incident and transmitted photon flux in ionization chambers I_0 and I_t, respectively. The Ru K-edge XAFS spectrum was recorded in transmission mode for standards such as Ru (99.9% metal basis, Aladdin) and RuO_2 (99.9% metal basis, Alfa Aesar Chemicals Co., Ltd), and fluorescence yield (FY) mode for the Ru/Mo_2TiC_2 and $Ru\text{-}NP/Mo_2TiC_2$

samples by measuring the Ru Kα X-ray emitted from the sample due to the low loading rate of Ru on MXene sheets. Standard Mo and Ru foils were used as references for beamline energy calibration at the Mo K-edge and Ru K-edge, respectively.

The background removal, normalization and Fourier transform (FT) of XAFS spectra were performed using Athena software. The extracted EXAFS data were weighted by k^3 and then converted to R space by FT to obtain the magnitude plots of the EXAFS spectra.

Photocatalytic and thermocatalytic measurements

Thermocatalytic and photothermal catalytic experiments were performed in the flow reactor, as shown in **Figure S22**. The catalyst powders (~3 mg) were dispersed in ethanol under sonication. Then, the suspension was transferred into a glass fiber filter with an area of 4.91 cm^2 through drop-casting, followed by drying the catalyst film in a vacuum oven. The reactor system comprised the flow controllers, reactor and online testing system. The reactor was equipped with a circular quartz window (r = 2.85 cm), which allowed illumination from the top. The temperature of the reactor is controlled by a homemade heating device with a thermocouple placed under the catalyst membrane. The reactor pressure was monitored by a digital manometer from Anchor Smart Instruments Ltd. Gas chromatography (Agilent 7890B) equipped with the thermal conductivity detection (TCD) and flame ionization device (FID) detectors was used to analyze the quantities of gaseous reactants and products online. The flow rates of feed gases were fixed at 5 mL/min CO_2, 5 mL/min H_2 and 10 mL/min N_2. In regard to photothermal RWGS experiments, a 300 W Xenon lamp was used as light source, without any filter or heating of the catalyst. After separation by gas chromatography, the concentrations of the different gases were analyzed using a thermal conductivity detection (TCD) device and a flame ionization device (FID) with a methanation unit.

$$F_{CO} = \frac{n(CO)}{m(cat) * \omega(Ru) * t}$$

$$F_{CH4} = \frac{n(CH_4)}{m(cat) * \omega(Ru) * t}$$

$$R_{CO2} = F_{CO} + F_{CH4}$$

The selectivity of CO is defined as

$$S(CO) = \frac{F(CO)}{F(CO) + F(CH4)}$$

where n is the yield of products (mol), ω(Ru) is the Ru loading in percent, m(cat) is the mass of the catalyst (g) and t is the irradiation time illuminated by the 300 W Xe arc lamp (h).

Isotope tracing experiments were performed using $^{13}CO_2$ (99.9 atomic % Sigma Aldrich) in a custom fabricated 1.5-mL stainless steel batch reactor with a fused silica view port sealed with Viton O-rings. The reactors were evacuated prior to being injected with $^{13}CO_2$ followed by H_2. The partial pressure was ~14 psi for both CO_2 and H_2. Isotope product gases were measured using an Agilent 7890A gas chromatographic mass spectrometer (GC-MS) with a 60-mGS-Carbon plot column fed to the mass spectrometer. The flow rates of feed gases were fixed at 5 mL/min CO_2, 5 mL/min H_2 and 10 mL/min N_2. A 300 W Xenon lamp was used as light source, without any filter or heating of the catalyst.

Temperature-programmed experiments: All temperature-programmed experiments, including CO_2, CO and H_2 temperature-programmed desorption (TPD) and temperature-programmed surface reaction (TPSR) for H_2-assisted CO activation, were performed in a flow reactor under atmospheric pressure. TPD experiments were performed on an automatic chemical adsorption instrument (FINETEC/FINE-SORB-3010). For CO-TPD, a 10 mg sample was put in a U-shaped quartz tube and flushed for 20 min with He (40 mL/min). Afterward, CO was introduced into the U-shaped quartz tube for 10 min for adsorption, followed by flushing with He (40 mL/min) for 10 min to remove the physical adsorption. Finally, the sample was heated to 700°C at

a rate of 10°C/min in He flow (40 mL/min). The desorbed CO was measured by a thermal conductivity detector (TCD). The temperature and current of TCD were 60°C and 90 mA, respectively.

For CO_2 and H_2-TPD, a 20 mg sample was fixed in a U-shaped quartz tube and flushed with Ar (40 mL/min) for 10 min, followed by heating to 300°C (10°C/min) for 60 min and then cooling to room temperature in the same Ar flow. Afterward, the sample was exposed to the adsorbate with a flow rate of 80 mL/min for 20 min at 25°C and then flushed with Ar flow (40 mL/min) for 10 min. Finally, the sample was heated to 600°C (10°C/min) in an Ar flow (40 mL/min). The temperature and current for TCD were 60°C and 90 mA, respectively.

Temperature-programmed surface reaction for H_2-assisted CO activation was performed in a quartz tube flow reactor with an inner diameter of 4 mm under atmospheric pressure. Twenty milligrams of catalyst was loaded into a reactor tube and held in place by quartz wool for each test. Before the reaction, the catalysts were pretreated under a 20 mL/min H_2/Ar mixture gas (1:1 ratio) at 500°C (10°C/min) for 2 h. Next, the inlet flow was first switched to 20 mL/min Ar for cooling to room temperature, and then the inlet flow was switched to 1% CO/Ar of 2 mL/min Ar of 18 mL/min. After flushing with the inlet flow for 10 min, the reactor was heated to 600°C at a 10°C/min heating rate. The CO was detected online using FTIR. To keep the mass fraction of active components of different catalysts constant, Ru/Mo_2TiC_2 and Ru-NP/Mo_2TiC_2 catalysts were diluted with commercial SiO_2.

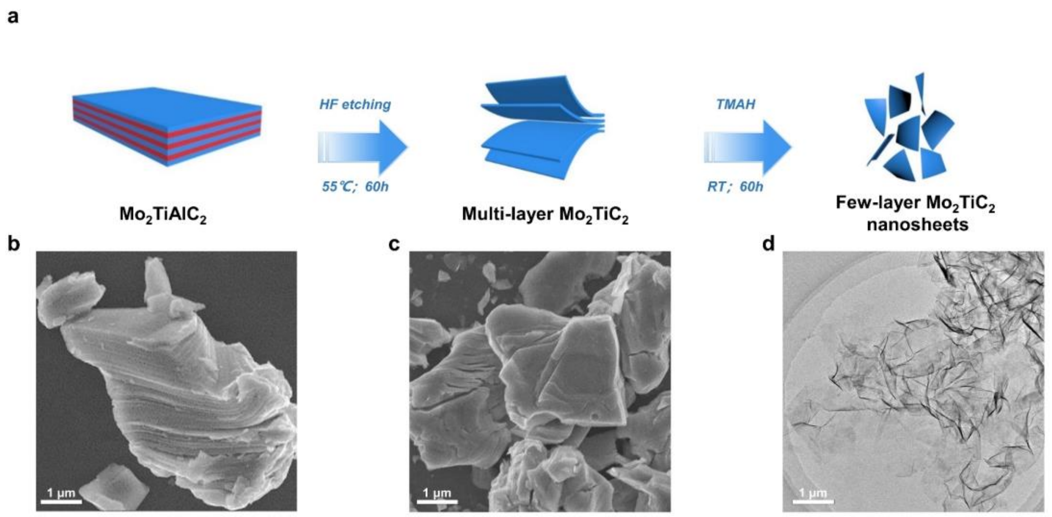

Figure S1. **a)** Schematic illustration of the preparation process of Mo_2TiC_2 MXene nanosheets. **b-c)** SEM image of the Mo_2TiAlC_2 precursor and multilayer Mo_2TiC_2 after etching Al layers in Mo_2TiAlC_2. **d)** TEM image of few-layer Mo_2TiC_2 MXene nanosheets.

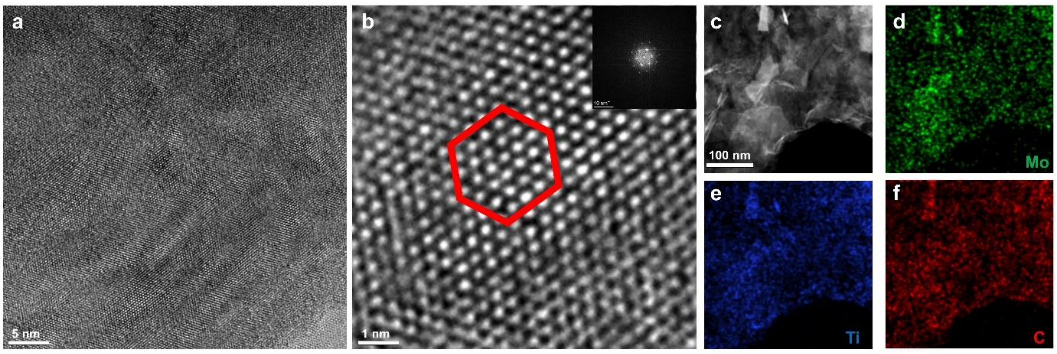

Figure S2. **a-b)** HRTEM image of few-layer Mo_2TiC_2 nanoplates (inset shows the fast Fourier transform (FFT) pattern extracted from the HRTEM image of few-layer Mo_2TiC_2 in **Figure S2b**). **c)** STEM image and **(d-f)** elemental mapping of few-layer Mo_2TiC_2 nanosheets. SEM images of the parent MAX phases (**Figure S1b**) and their MXenes (**Figure S1c**) indicate the transformation from 3D bulk structure to 2D accordion-like nanoflakes. After the intercalation process of Mo_2TiC_2 nanosheets in tetramethylammonium hydroxide (TMAH), few-layer Mo_2TiC_2 nanosheets with ordered hexagonal distribution ($P6_3/mmc$) can be synthesized (**Figure S2a-b**). Energy-dispersive X-ray (EDX) spectroscopy elemental analysis further demonstrates the homogenous distribution of Mo, C and Ti throughout the ultrathin Mo_2TiC_2 nanosheets (**Figure S2c-f**).

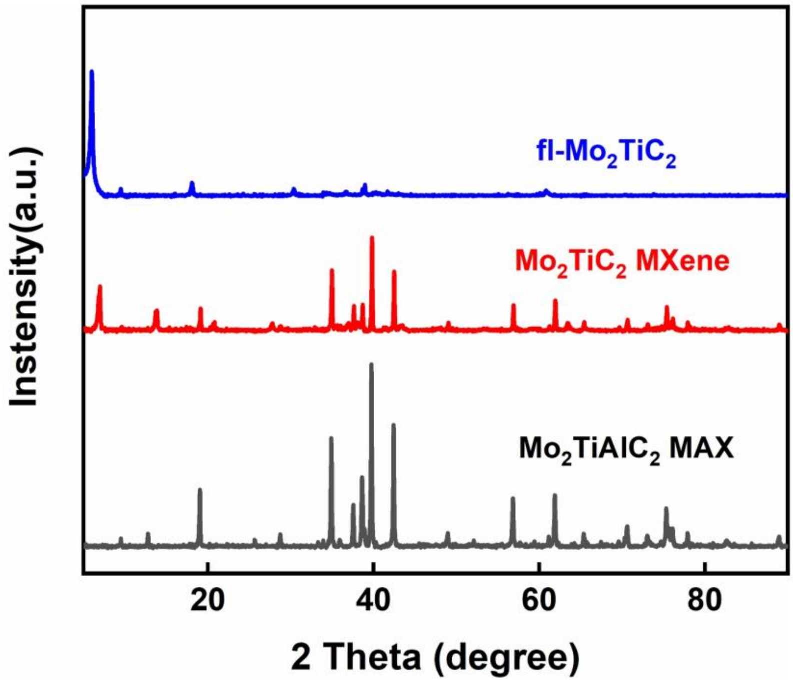

Figure S3. XRD patterns of the Mo_2TiAlC_2 precursor, multilayer Mo_2TiC_2 after etching the Al layers, and few-layer Mo_2TiC_2 nanosheets. In contrast with the Mo_2TiAlC_2 precursor, these characteristic peaks belonging to $Mo_2TiC_2T_x$ after etching and delamination (middle red and top blue lines) downshifted to lower angles, mainly resulting from an increase in the c lattice parameter from 18.59 Å to 25.39 and 29.59 Å, respectively. These results indicate the successful etching and exfoliation process of few-layer Mo_2TiC_2 nanosheets.

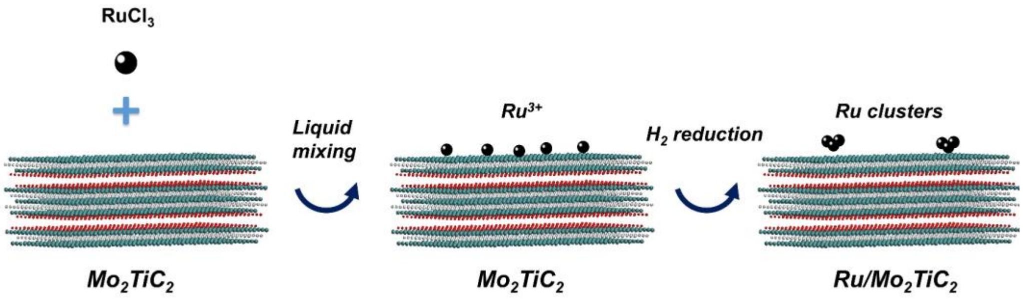

Figure S4. Schematic illustration of the preparation processes of Ru/Mo_2TiC_2 catalysts.

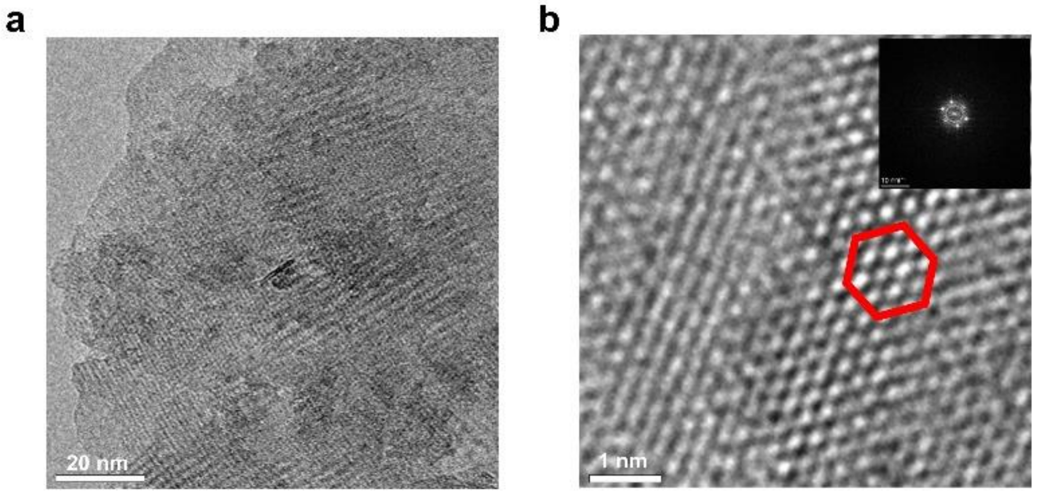

Figure S5. **a)** TEM image of Ru/Mo_2TiC_2. **b)** HRTEM image of Ru/Mo_2TiC_2 (inset shows the fast Fourier transform (FFT) pattern extracted from the HRTEM image of Ru/Mo_2TiC_2 in **Figure S5b**).

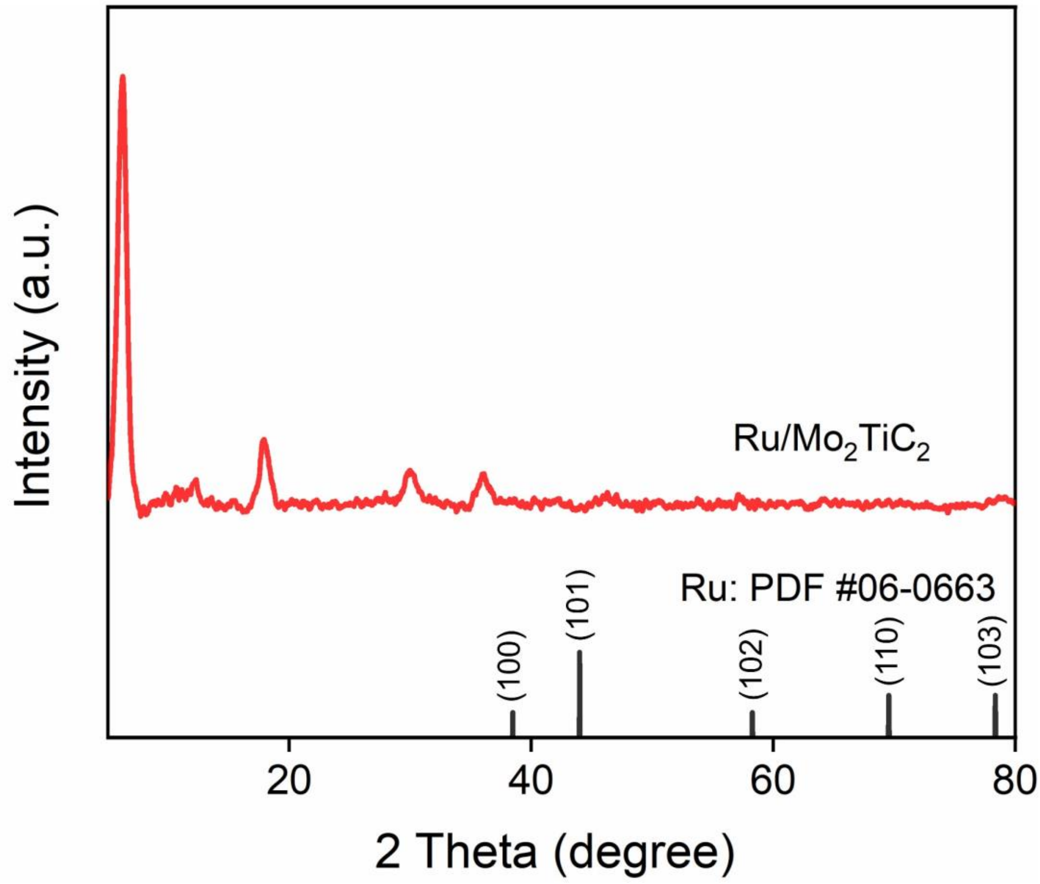

Figure S6. XRD pattern of Ru/Mo_2TiC_2.

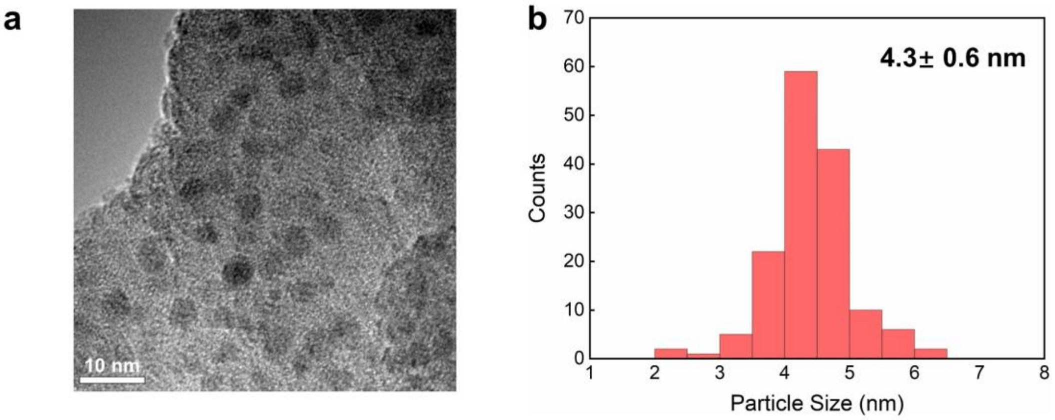

Figure S7. **a)** TEM image of Ru-NP/SiO_2-1. **b)** Size distribution of Ru nanoparticles in Ru-NP/SiO_2-1.

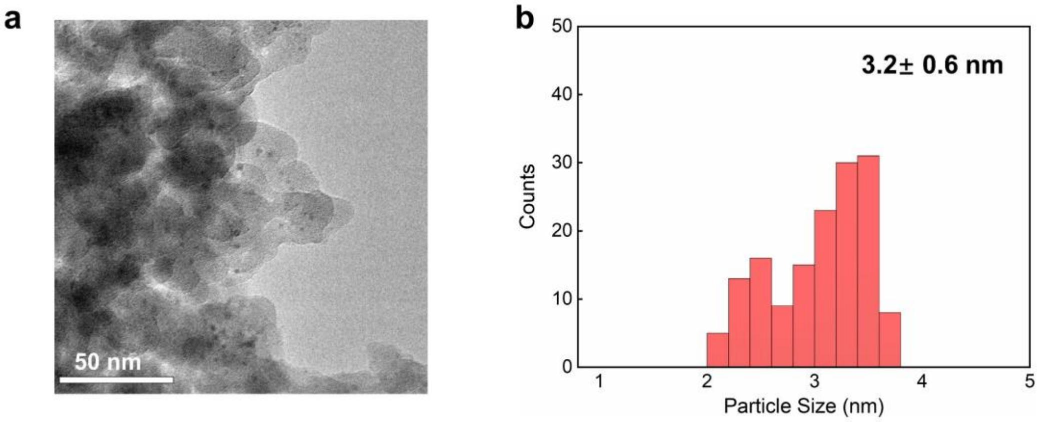

Figure S8. **a)** TEM image of Ru-NP/SiO_2-2. **b)** Size distribution of Ru nanoparticles in Ru-NP/SiO_2-2.

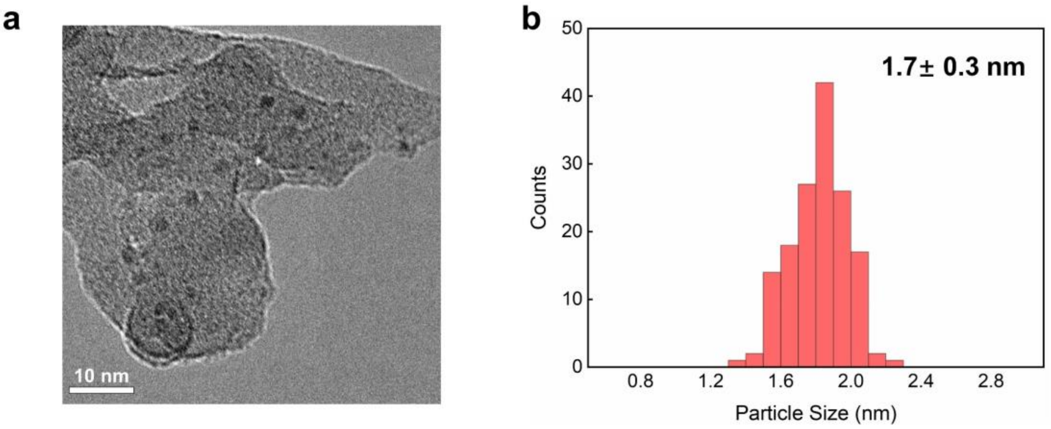

Figure S9. **a)** TEM image of Ru/SiO_2. **b)** Size distribution of Ru nanoparticles in Ru/SiO_2.

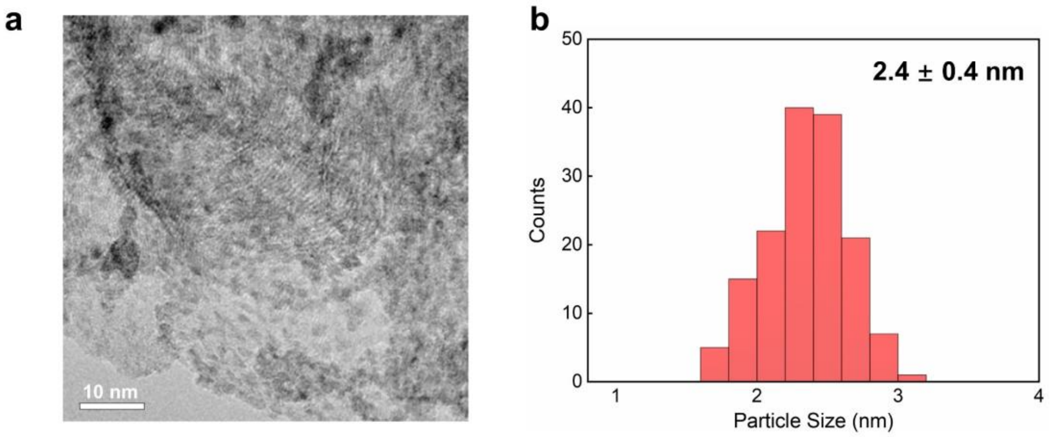

Figure S10. **a)** TEM image of Ru-NP/Mo_2TiC_2. **b)** Size distribution of Ru nanoparticles in Ru-NP/Mo_2TiC_2.

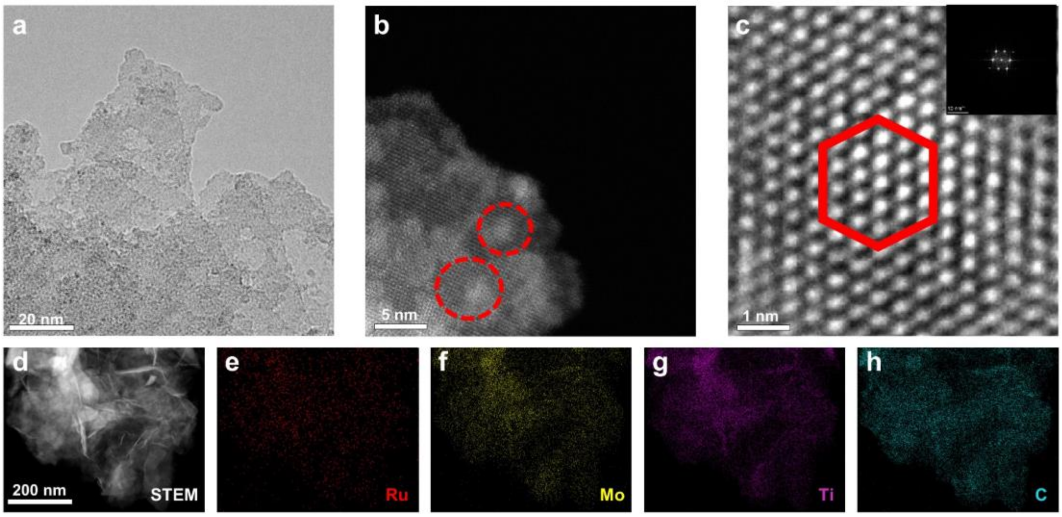

Figure S11. **a)** TEM image of Ru-NP/Mo_2TiC_2. **b)** Aberration-corrected HAADF-STEM image of Ru-NP/Mo_2TiC_2. **c)** HRTEM image of few-layer Mo_2TiC_2 nanosheets (inset shows the fast Fourier transform (FFT) pattern extracted from the HRTEM image of Mo_2TiC_2 in **Figure S11c**). **d)** STEM image and **e-h)** elemental mapping images of Ru-NP/Mo_2TiC_2. It shows that the introduction of Ru nanoparticles does not change the morphology and crystalline structure of few-layer Mo_2TiC_2 nanosheets. Energy-dispersive X-ray (EDX) spectroscopy elemental analysis in **d-h** further demonstrates the homogenous distribution of Ru nanoparticles (average size of ~2.4 nm) on ultrathin Mo_2TiC_2 nanosheets.

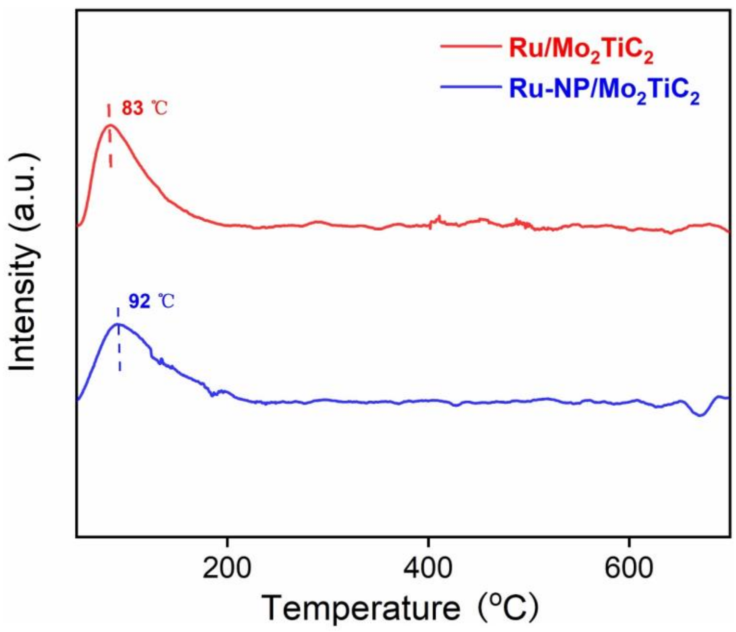

Figure S12. CO-TPD profiles of Ru/Mo_2TiC_2 and $Ru\text{-}NP/Mo_2TiC_2$ catalysts.

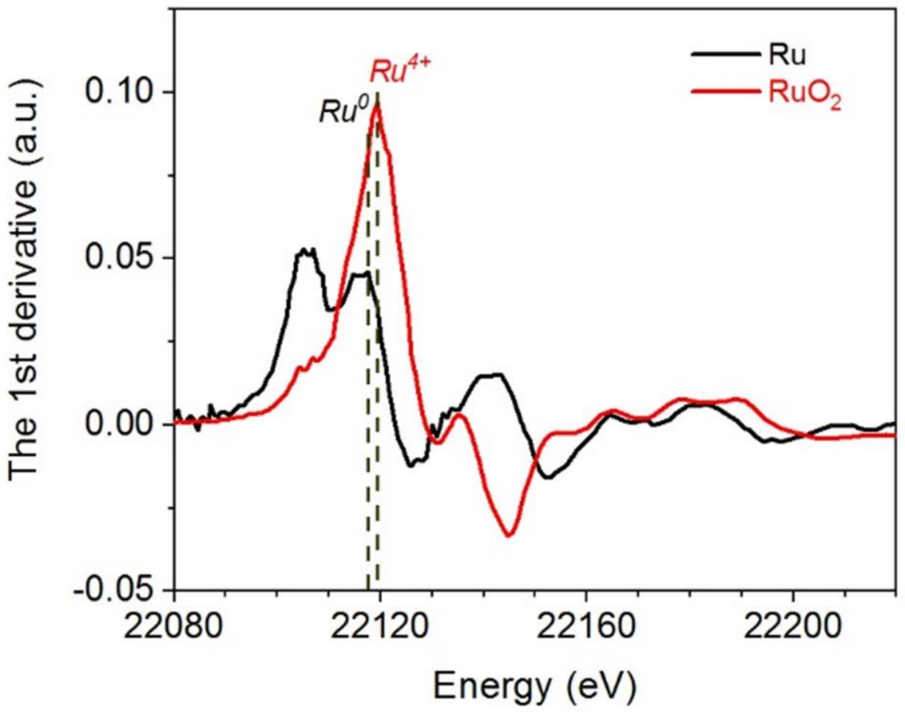

Figure S13. The feature used to determine the K-edge position of Ru foil and RuO_2 determined by the first derivative of normalized spectra at the Ru-K edge of Ru and RuO_2 foil references.

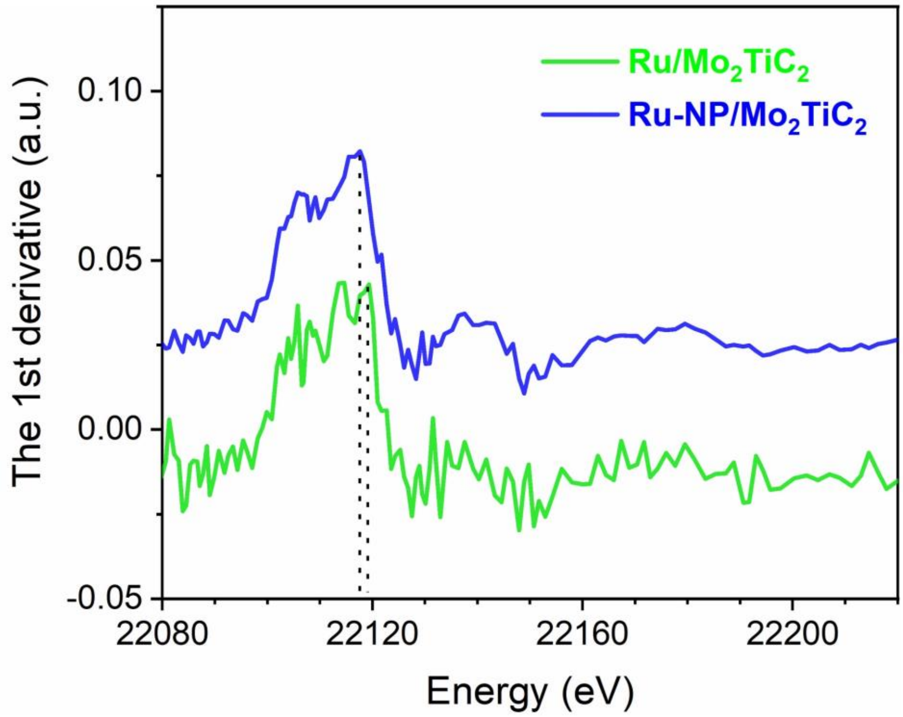

Figure S14. The feature used to determine the K-edge position of MXene-based catalysts determined by the first derivative of normalized spectra at the Ru K-edge of Ru/Mo_2TiC_2 and Ru-NP/Mo_2TiC_2. The X-ray absorption near-edge spectroscopy (XANES) spectra show that the adsorption edges of Ru/Mo_2TiC_2 and Ru-NP/Mo_2TiC_2 samples were located between Ru foil and RuO_2, indicating the slightly positive valence state of Ru species on Mo_2TiC_2 nanosheets (**Figure 2b**). An important and common application of XANES is to use the shift of the edge position to determine the valence state. With good reference spectra, the oxidation state of the element of interest can be determined by the position of the absorption energy threshold with very good precision and reliability. Thus, it is reported that the oxidation state of Ru species in Ru/Mo_2TiC_2 could be obtained from the edge position of Ru in K-edge XANES. Based on the metallic Ru and RuO_2 samples (the edge positions of 22117.2 and 22119.3 eV correspond to Ru chemical states of 0 and +4, respectively), the average oxidation states of Ru in Ru/Mo_2TiC_2 and Ru-NP/Mo_2TiC_2 are +2.19 and +0.76, respectively (Figures S12-S13).

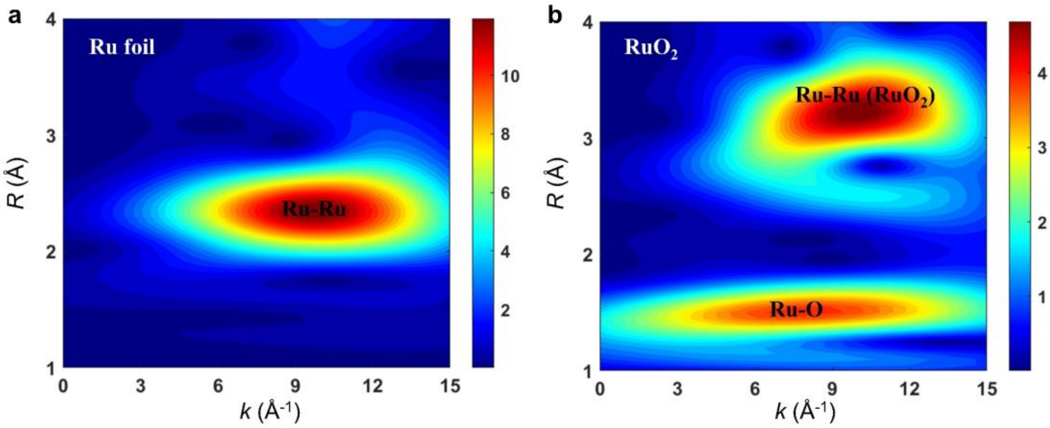

Figure S15. Wavelet transformation of the Ru K-edge EXAFS signals of Ru foil and RuO_2 references based on Morlet wavelets with optimum resolutions at the first and higher coordination shells. The wavelet transformation (WT) of the Ru K-edge EXAFS signals of Ru/Mo_2TiC_2 and Ru-NP/Mo_2TiC_2 is demonstrated in **Figure 2e-f**. For the Ru foil, there is a characteristic hotspot at 8.70 $Å^{-1}$ (*k* value) with R values of 2.38 Å, which is attributed to the Ru-Ru scattering pairs (**Figure S15a**). Simultaneously, the WT contour plots exhibited another two hotspots at R=1.53 Å, *k*=9.02 $Å^{-1}$ and R=3.20 Å, *k*=10.18 $Å^{-1}$, ascribed to Ru-O and Ru-Ru (RuO_2) scattering pairs, respectively (**Figure S15b**). Compared with the reference materials, there are two respective hotspots observed at R=2.38 Å, *k*=8.70 $Å^{-1}$ and R=1.53 Å, *k*=9.02 $Å^{-1}$ of Ru/Mo_2TiC_2 and Ru-NP/Mo_2TiC_2, which are well consistent with the reference materials. Moreover, the intensity maximum of Ru/Mo_2TiC_2 at the Ru-O scattering pairs is obviously enhanced as compared with that in Ru-NP/Mo_2TiC_2, indicative of the higher oxidation state of Ru in the Ru/Mo_2TiC_2 catalyst. The relative intensity of these two peaks further confirmed the higher oxidation state of Ru in Ru/Mo_2TiC_2. This difference becomes more evident from the wavelet transform of the EXAFS spectra.

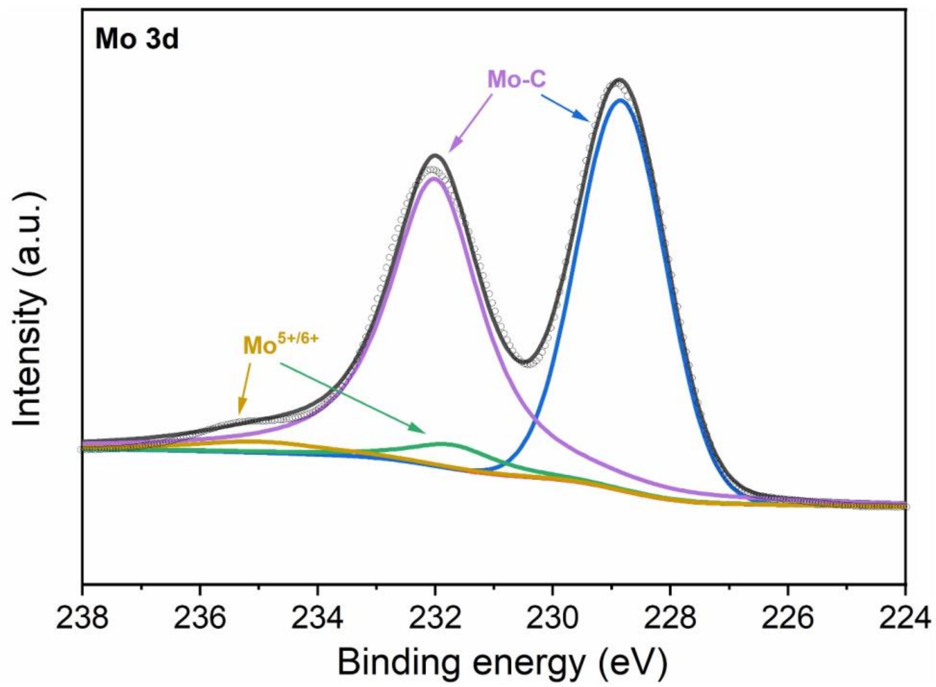

Figure S16. High-resolution Mo 3d XPS spectra of Mo_2TiC_2.

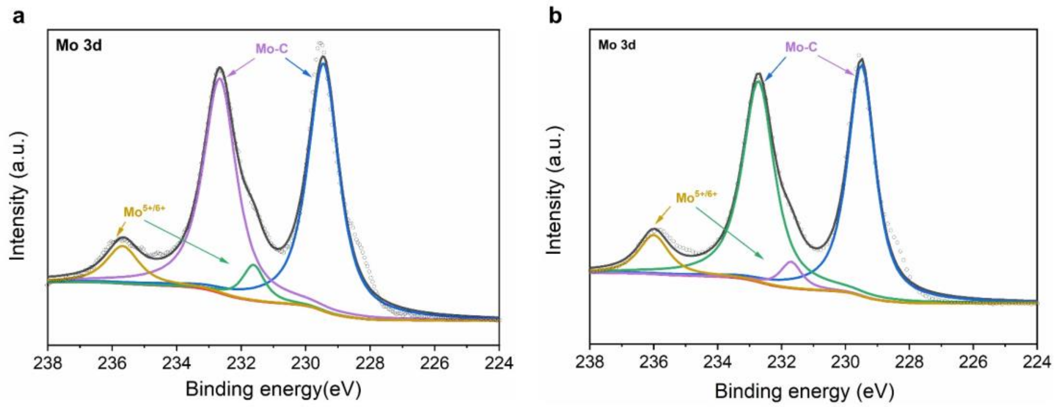

Figure S17. High-resolution Mo 3d XPS spectra of **(a)** Ru/Mo_2TiC_2 and **(b)** Ru-NP/Mo_2TiC_2. To investigate the interface in the Ru-Mo_2TiC_2 MXene system, we first analyzed the chemical state of the catalysts by X-ray photoelectron spectroscopy (XPS). The corresponding C 1s and Ru 3d spectra of Ru/Mo_2TiC_2 and Ru-NP/Mo_2TiC_2 are shown in **Figure 2a**. A binding energy peak was observed for both samples at approximately 280.6 eV. This peak was assigned to an oxidation state of Ru ($Ru^{\delta+}$) between Ru^0 and Ru^{4+} owing to the strong electronic interaction between Ru nanoparticles and few-layer Mo_2TiC_2 MXene materials. Moreover, the corresponding peak belonging to $Ru^{\delta+}$ of Ru/Mo_2TiC_2 exhibited a positive shift compared to Ru-NP/Mo_2TiC_2, which is indicative of the higher oxidation states in the Ru/Mo_2TiC_2 catalyst. Meanwhile, in contrast to pure Mo_2TiC_2 nanosheets, the peak positions of both Ru/Mo_2TiC_2 and Ru-NP/Mo_2TiC_2 samples shifted to higher binding energies, representing higher oxidation states of Mo due to the introduction of Ru species.

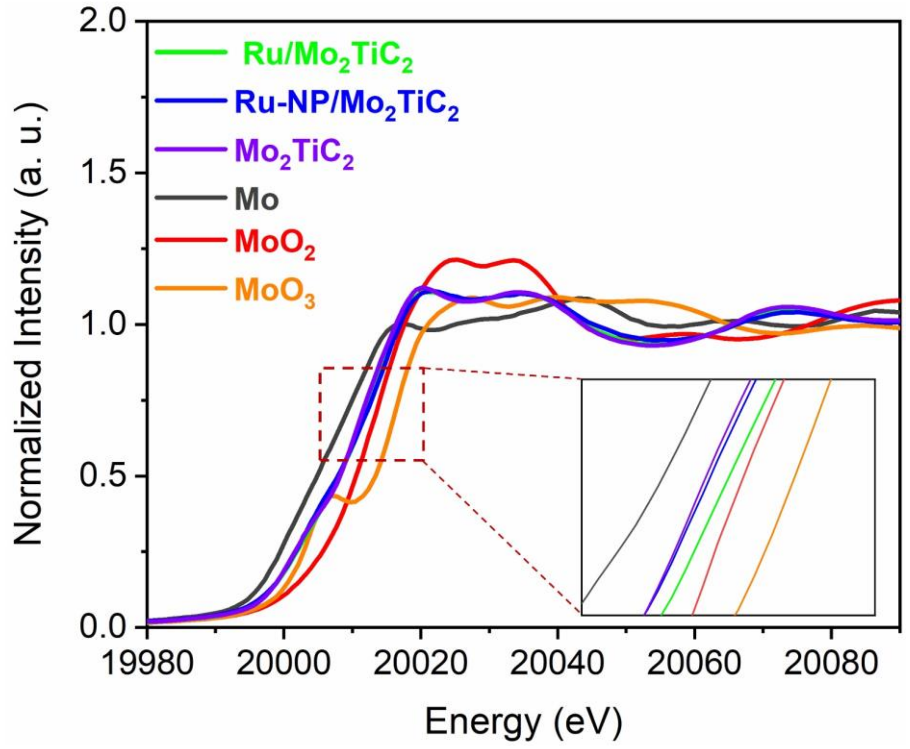

Figure S18. Normalized XANES spectra at the Mo K-edge of Mo foil, MoO_2, MoO_3, $Mo_2TiC_2T_x$, Ru/Mo_2TiC_2 and $Ru\text{-}NP/Mo_2TiC_2$.

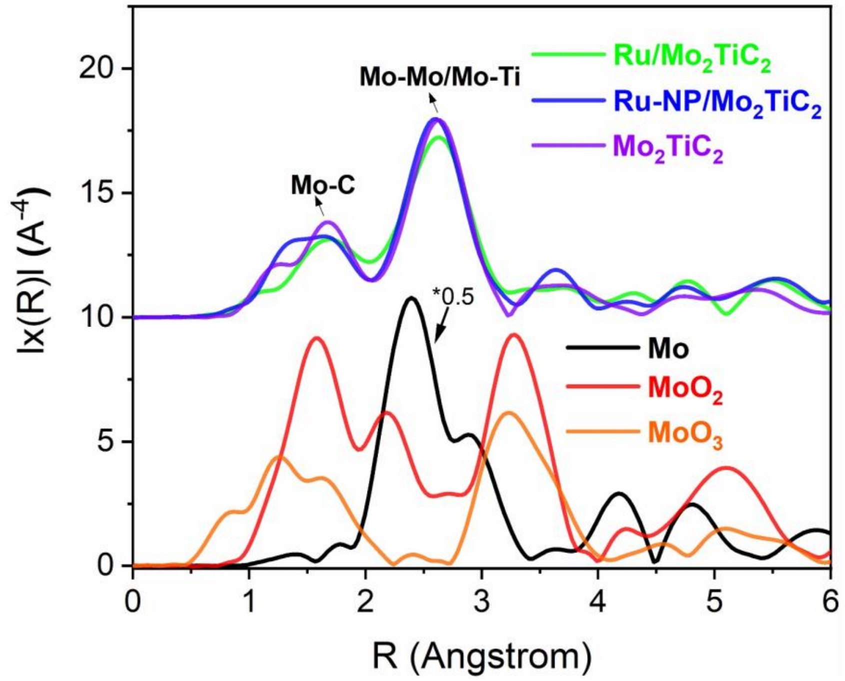

Figure S19. The corresponding FT-EXAFS spectra derived from the Mo K-edge of Mo foil, MoO_2, MoO_3, $Mo_2TiC_2T_x$, Ru/Mo_2TiC_2 and Ru-NP/Mo_2TiC_2. The XANES patterns of Ru/Mo_2TiC_2 and few-layer Mo_2TiC_2 nanosheets are similar, demonstrating that the introduction of Ru nanoparticles did not change the morphology and crystalline structure of few-layer Mo_2TiC_2 nanosheets. The Mo K-edge position of Mo_2TiC_2, Ru/Mo_2TiC_2 and Ru-NP/Mo_2TiC_2 were clearly located between the Mo foil and MoO_3 references, indicating different valence states of Mo species in Mo_2TiC_2 MXene materials.

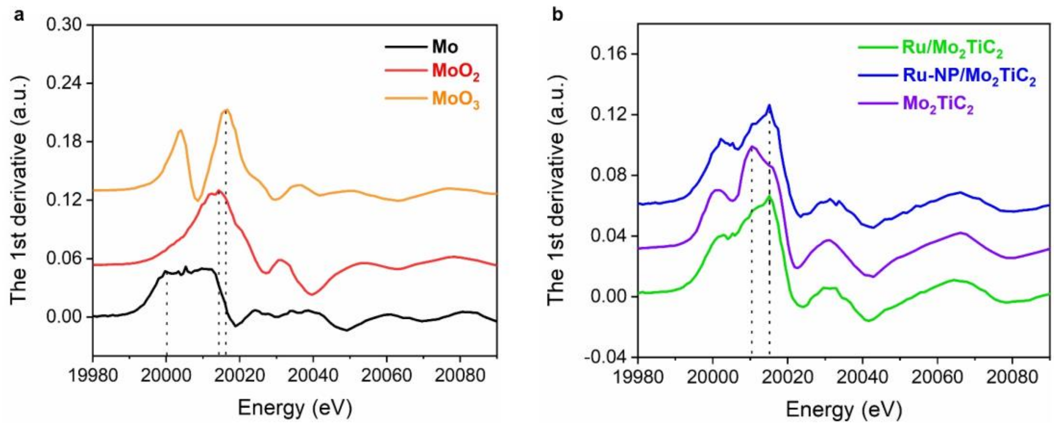

Figure S20. The feature used to determine the K-edge position of MXene-based catalysts was determined by the first derivative of normalized spectra at the Mo-K edge of Mo, MoO_2, MoO_3, Mo_2TiC_2, Ru/Mo_2TiC_2 and Ru-NP/Mo_2TiC_2.

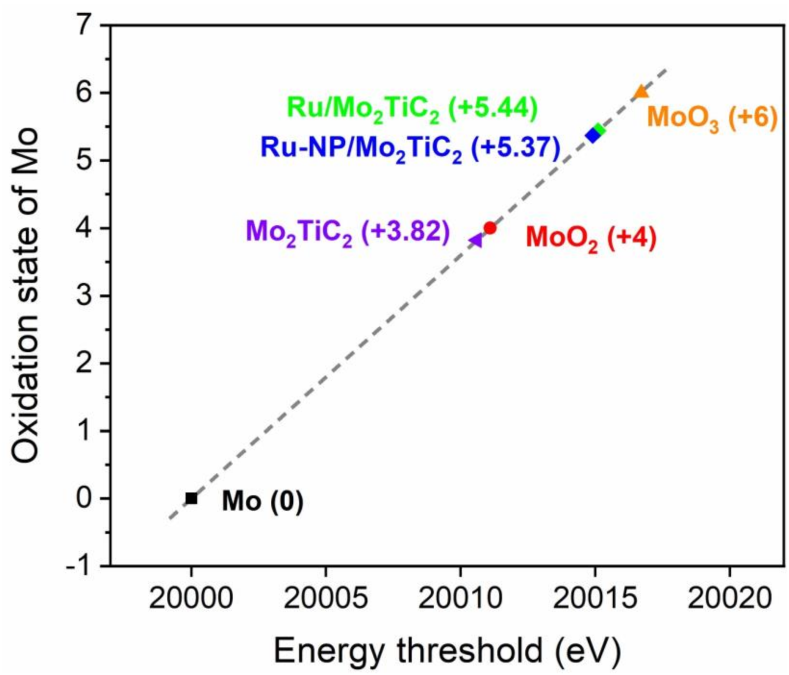

Figure S21. The oxidation states of molybdenum in the reference and synthesized materials determined from the edge position in Mo K-edge XANES spectra. In view of the aforementioned method to determine the valence state calculated by the first derivative of normalized spectra, the oxidation state of Mo species in all samples can be determined. According to the references of Mo foil, MoO_2 and MoO_3 (edge positions 20000.4, 20014.18, and 20016.57 eV represent Mo oxidation states of 0, +4, and +6, respectively, **Figure S20a**), the average valence state of Mo in pristine Mo_2TiC_2 nanosheets is +3.82, resulting from existing surface terminal groups during the etching and delamination process of the Mo_2AlTiC_2 MAX phase. When introducing Ru nanoparticles on the Mo_2TiC_2 nanosheets, Ru/Mo_2TiC_2 and Ru-NP/Mo_2TiC_2 samples exhibited higher oxidation states of Mo species than pristine Mo_2TiC_2 nanosheets. In detail, the average oxidation states of Mo in Ru/Mo_2TiC_2 and Ru-NP/Mo_2TiC_2 were +5.44 and +5.37, respectively (**Figure S20b and S21**).

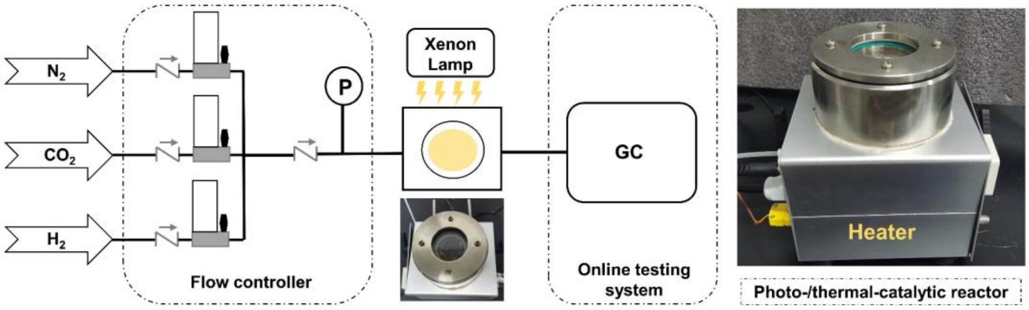

Figure S22. Photograph of the flow reactor for photothermal catalytic and thermocatalytic testing.

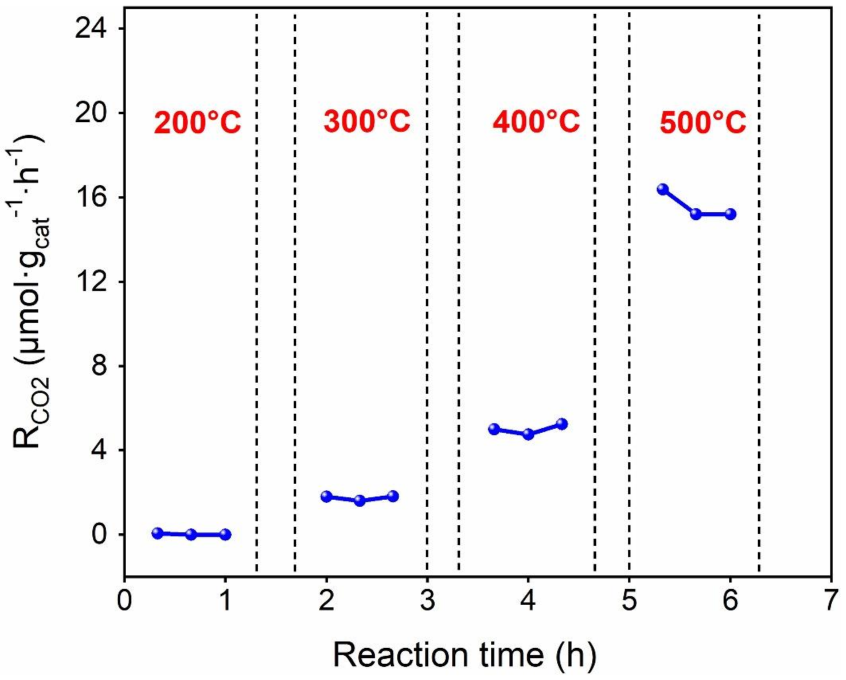

Figure S23. Temperature dependent performance of Mo_2TiC_2 in thermocatalytic CO_2 hydrogenation tested in the flow reactor.

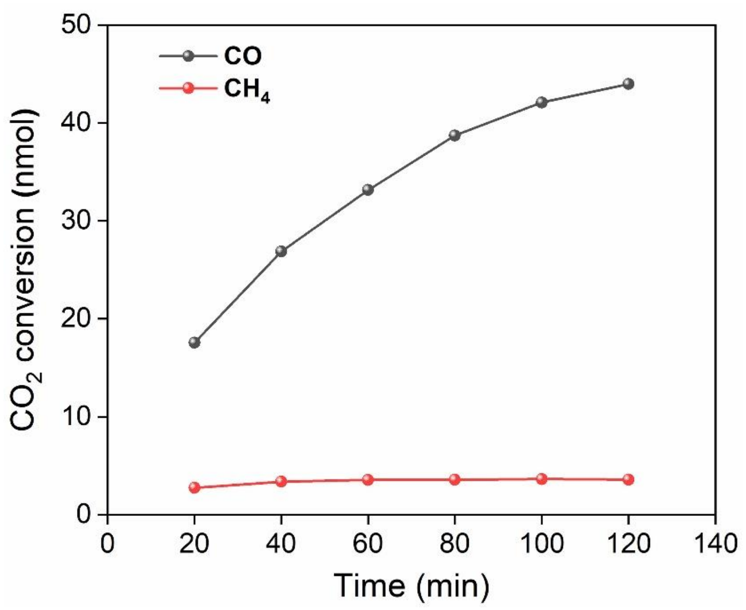

Figure S24. Time-course photothermal conversion of CO_2 by as-obtained Mo_2TiC_2 nanosheets in the batch reactor. Reaction conditions: 15 mg of catalysts, H_2/CO_2=1:1, total pressure of 1 atm, and under the simulated sunlight intensity of 3.0 W/cm^2.

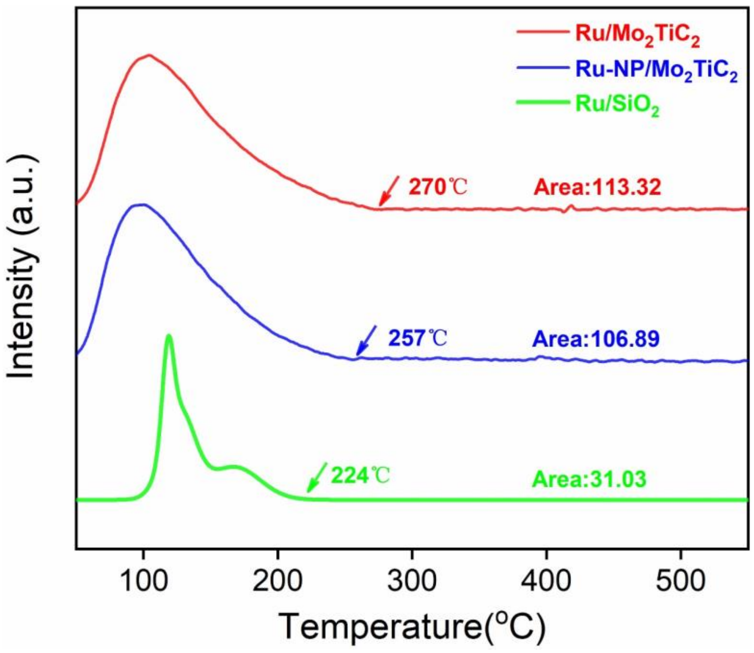

Figure S25. CO_2-TPD profiles of Ru/Mo_2TiC_2, Ru-NP/Mo_2TiC_2 and Ru/SiO_2 catalysts.

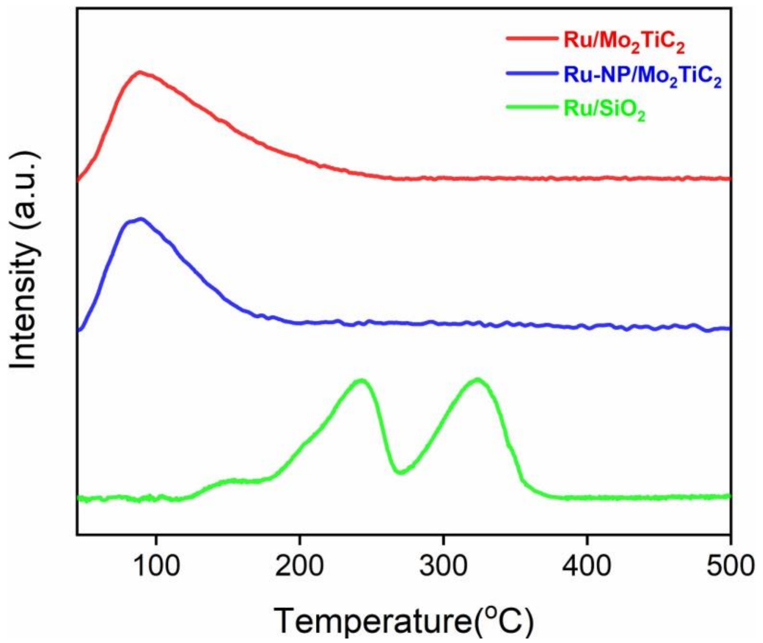

Figure S26. H_2-TPD profiles of Ru/Mo_2TiC_2, Ru-NP/Mo_2TiC_2 and Ru/SiO_2 catalysts. Additionally, our results indicated that Ru/SiO_2 exhibited the highest activity below 500℃ among the three catalysts. Correspondingly, H_2 and CO_2-TPD analysis were performed to verify enhanced activity of Ru/SiO_2. It is shown that Ru/Mo_2TiC_2, Ru-NP/Mo_2TiC_2 and Ru/SiO_2 catalyst showed weak adsorption ability of CO_2. The H_2-TPD analysis, however, indicated stronger H_2 adsorption of Ru/SiO_2 than Mo_2TiC_2 MXene supported Ru catalysts, which is an obvious indicator of higher thermocatalytic activity among the three catalysts.

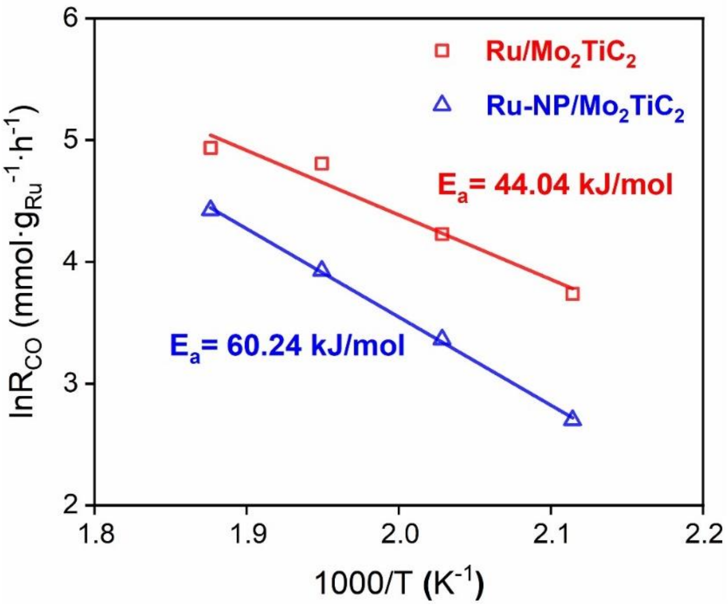

Figure S27. Pseudo-Arrhenius plots drawn from temperature-dependent measurements of CO rates between 200 and 280℃. The apparent activation energy for the RWGS reaction is indicated in the plots.

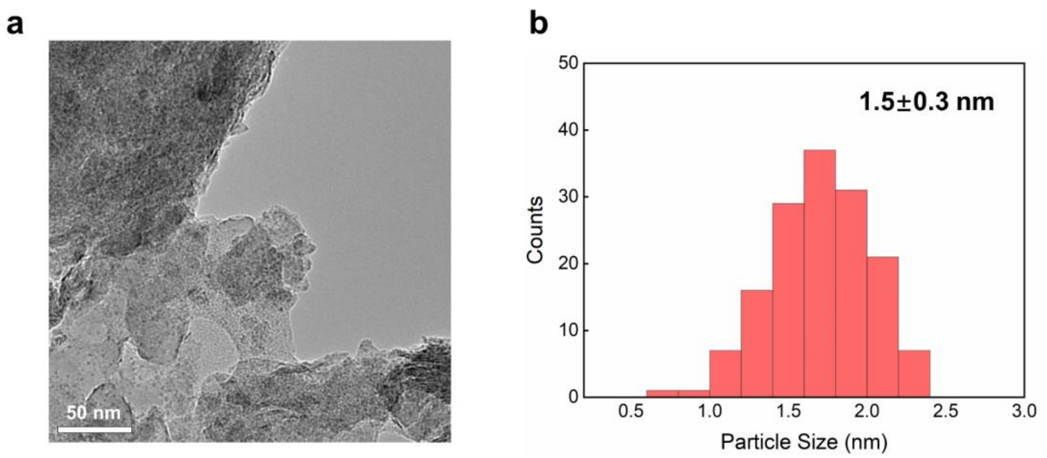

Figure S28. **a)** TEM image of the Ru/Mo_2TiC_2 catalyst after a continuous 15-h run at 500 ℃. **b)** Size distribution of Ru nanoparticles in the tested Ru/Mo_2TiC_2 catalyst.

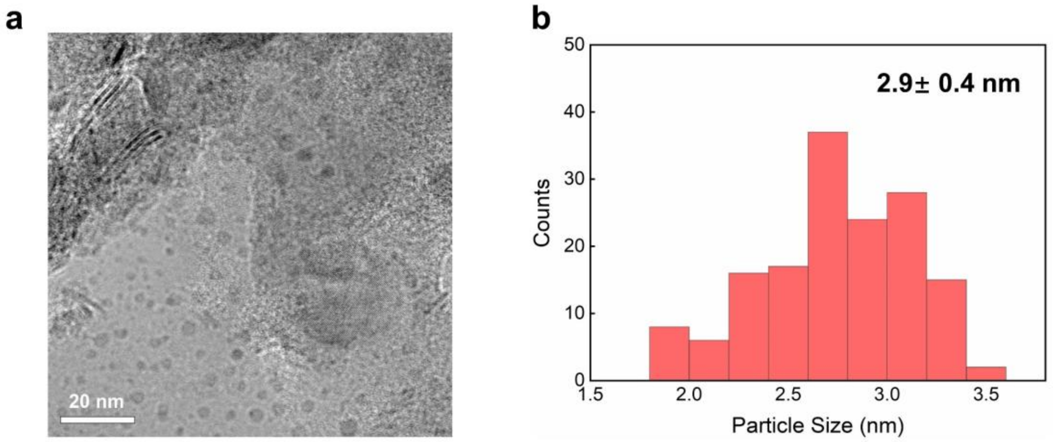

Figure S29. **a)** TEM image of the Ru-NP/Mo_2TiC_2 catalyst after a continuous 15-h run at 500℃. **b)** Size distribution of Ru nanoparticles in the tested Ru-NP/Mo_2TiC_2 catalyst.

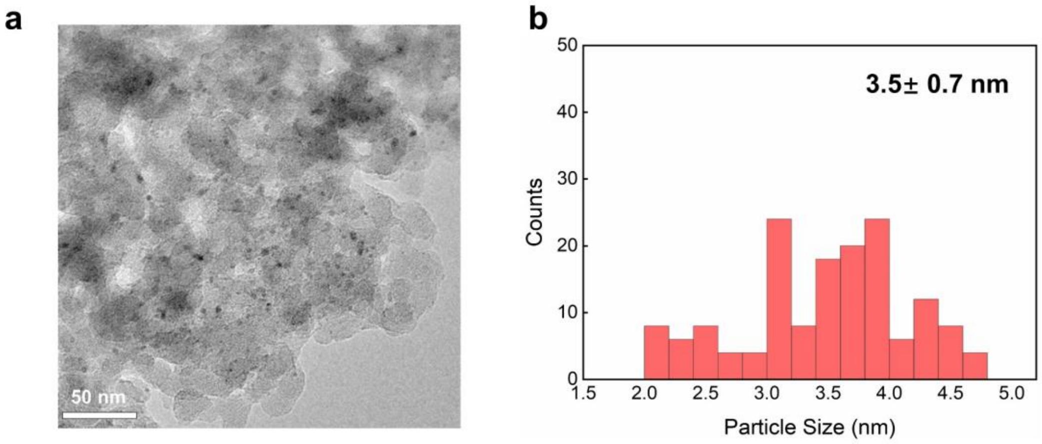

Figure S30. **a)** TEM image of the Ru/SiO_2 catalyst after a continuous 15-h run at 500℃. **b)** Size distribution of Ru nanoparticles in the tested Ru/SiO_2 catalyst.

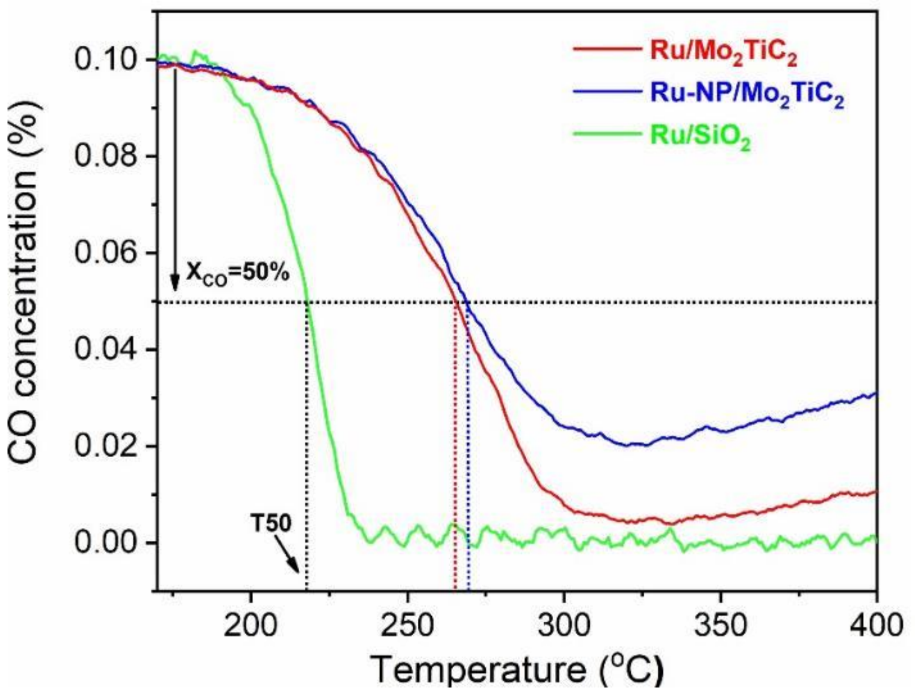

Figure S31. CO signal of H_2-assisted CO hydrogenation TPSR for Ru/Mo_2TiC_2, Ru-NP/Mo_2TiC_2 and Ru/SiO_2 catalysts.

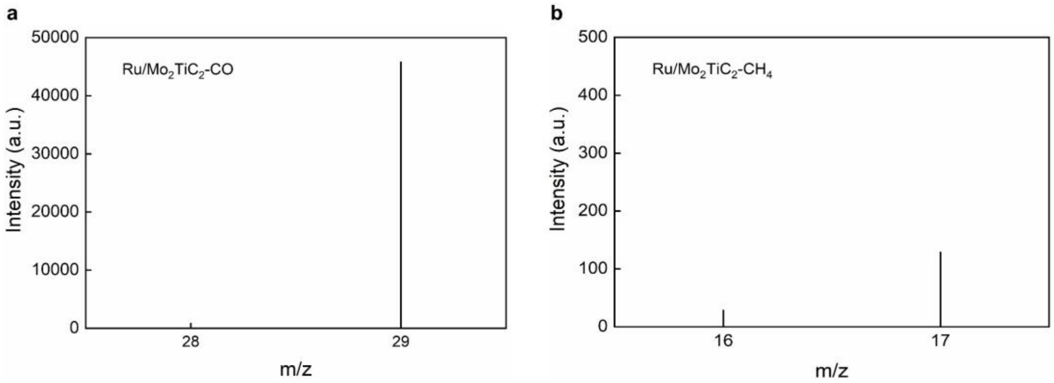

Figure S32. Typical mass spectra (m/z=28-29, 16-17) of products of **a)** ^{13}CO and **b)** $^{13}CH_4$ when using the isotope-labelled $^{13}CO_2$ as the reactant based on the Ru/Mo_2TiC_2 catalyst.

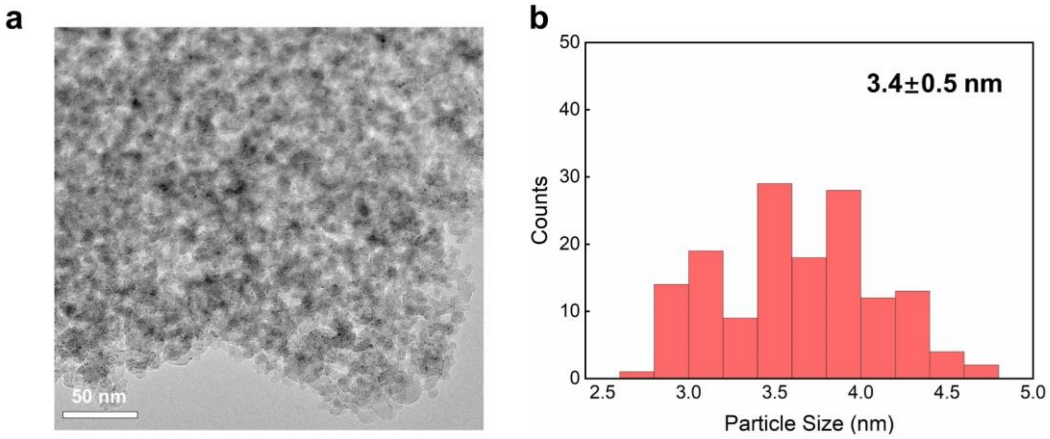

Figure S33. **a)** TEM image of the Ru/SiO_2 catalyst after catalyzing the hydrogenation of CO_2 under the illumination of 3.8 W/cm^2 in the flow reactor. **b)** Size distribution of Ru nanoparticles in the tested Ru/SiO_2 catalyst.

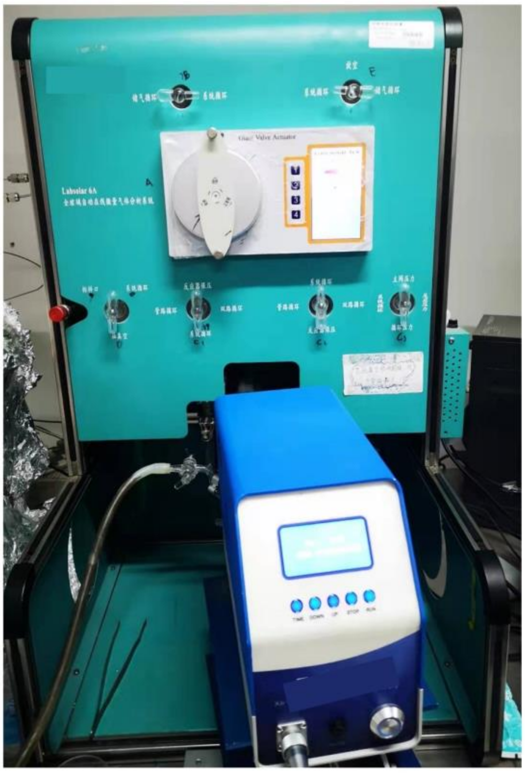

Figure S34. Photograph of the batch reactor system (Beijing Perfectlight, Labsolar 6A).

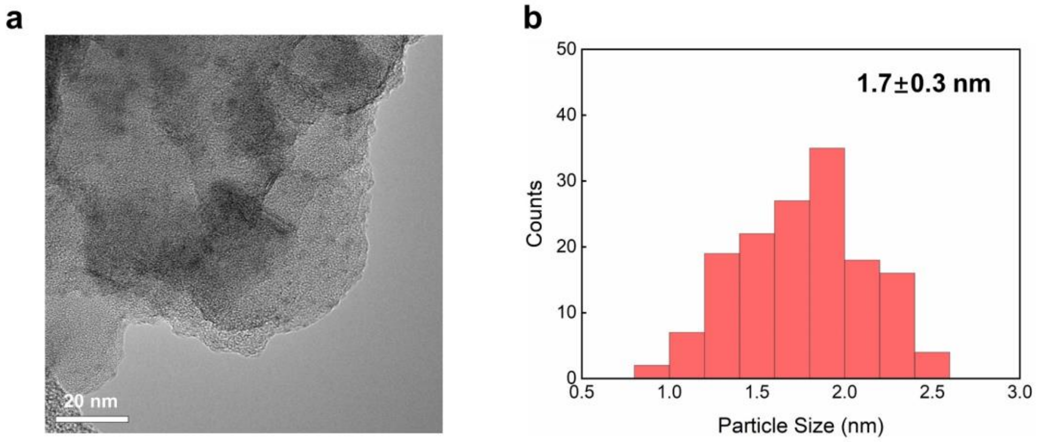

Figure S35. **a)** TEM image of the Ru/Mo_2TiC_2 catalyst after a continuous10-h photothermal catalytic test under a light intensity of 3.4 W/cm^2. **b)** Size distribution of Ru nanoparticles in the tested Ru/Mo_2TiC_2 catalyst.

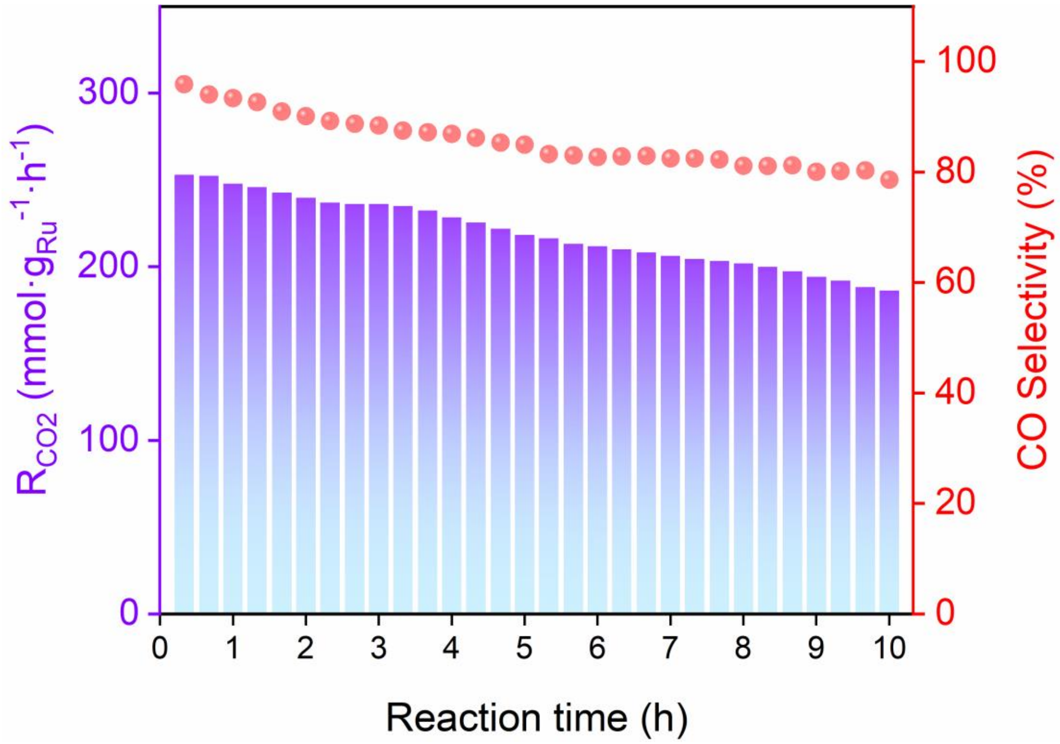

Figure S36. Photothermal catalytic performance of Ru-NP/Mo_2TiC_2 in catalyzing the hydrogenation of CO_2 under the illumination of 3.4 W/cm^2 for a continuous 10-hour test in the flow reactor.

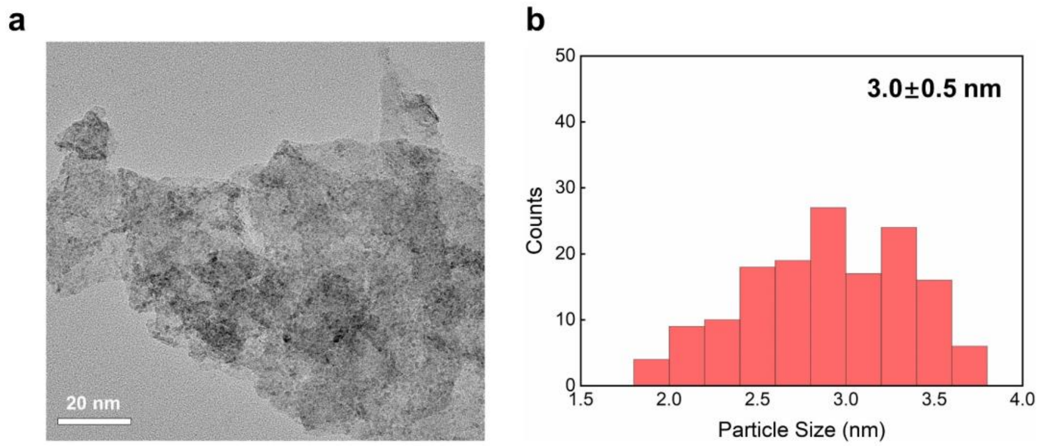

Figure S37. **a)** TEM image of the Ru-NP/Mo_2TiC_2 catalyst after a continuous10-h photothermal catalytic test under a light intensity of 3.4 W/cm^2. **b)** Size distribution of Ru nanoparticles in the tested Ru-NP/Mo_2TiC_2 catalyst.

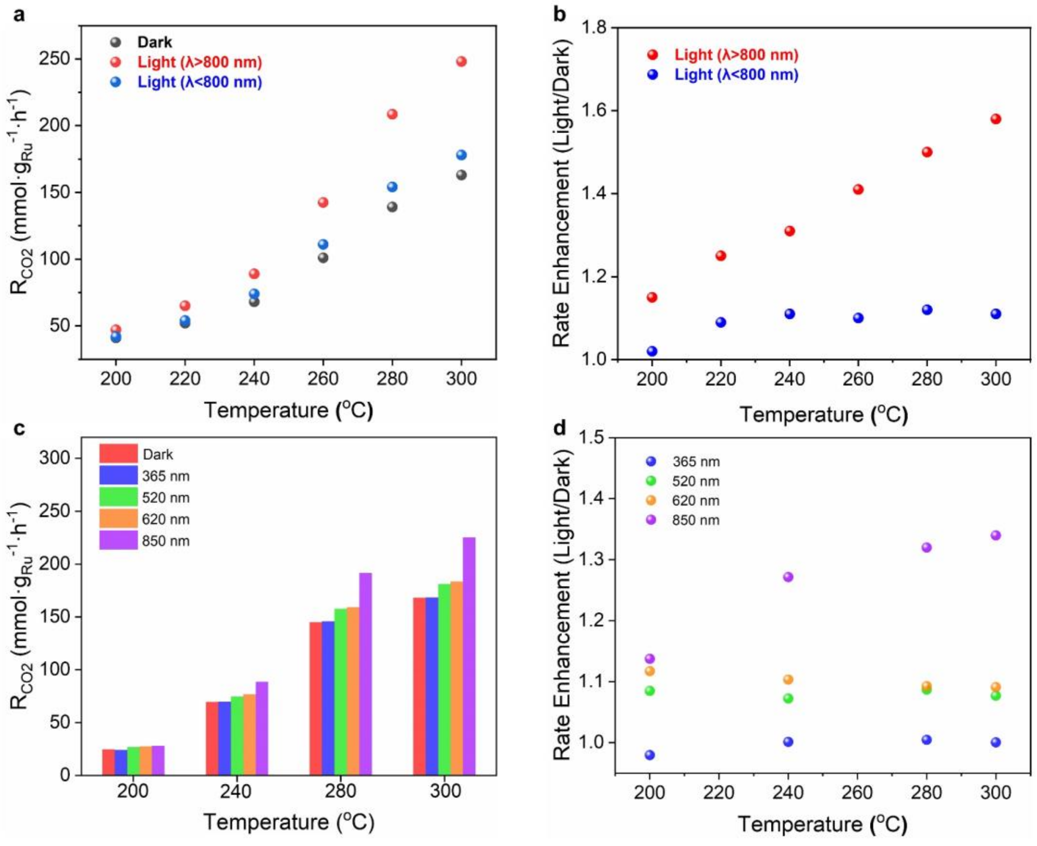

Figure S38. **a)** Reaction rate of CO_2 production on the Ru/Mo_2TiC_2 catalyst as a function of temperature under purely thermal conditions and under light illumination (λ>800 nm: the near infrared region range; λ<800 nm: the ultraviolet−visible light region; the light intensity of 0.36 W/cm^2). **b)** Rate enhancement (CO_2 production rate ratio of light vs. purely thermal conditions) as a function of temperature. **c)** Reaction rate of CO_2 production on the Ru/Mo_2TiC_2 catalyst as a function of temperature under purely thermal conditions and under different wavelengths of monochromatic light sources. **d)** Rate enhancement (CO_2 production rate ratio of the Ru/Mo_2TiC_2 catalyst under different wavelengths of monochromatic light sources vs. purely thermal conditions) as a function of temperature.

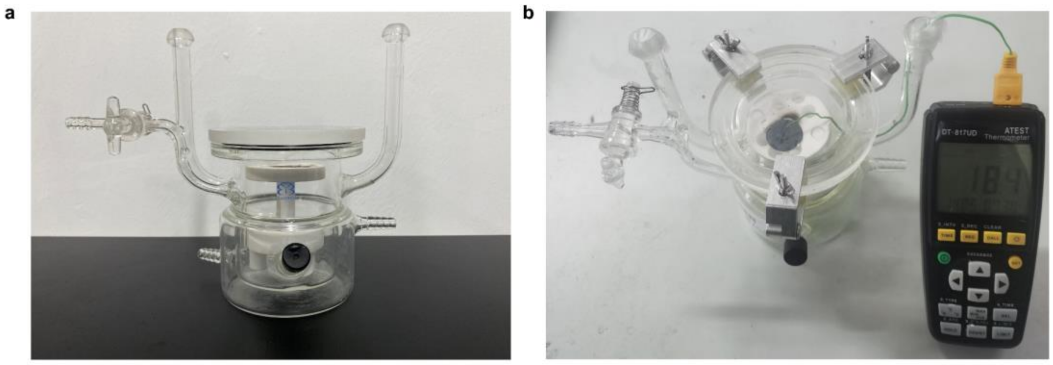

Figure S39. **a)** Photograph of the quartz reactor. **b)** Enlarged image of the reactor and the temperature sensor. A directly-contacting thermocouple equipped with the temperature sensor (ATEST, DT-817UD) was used to probe the real-time surface temperature of the catalyst under the condition of reactions after air-tightness test.

Table S1. Summary of Ru catalysts with different supports.

Sample	Support	Loading of Ru (wt %)[a]	Pretreatment conditions in H_2	Size of Ru (nm)[b]
Ru/Mo_2TiC_2	Mo_2TiC_2	2.1	500°C	1.2
Ru-NP/SiO_2-1	SiO_2	2.0	500°C	4.3
Ru-NP/SiO_2-2	SiO_2	0.5	500°C	3.2
Ru/SiO_2	SiO_2	0.5	200°C	1.7
Ru-NP/Mo_2TiC_2	Mo_2TiC_2	7.8	500°C	2.4

[a]*Determined by ICP-MS.* [b]*Obtained by measuring 150 Ru particles in TEM images.*

Table S2. Ru dispersity and particle size of different catalysts in catalytic reactions.

Sample	Support	Loading of Ru (wt %)[a]	Ru dispersion (%)[b]	Size of Ru (nm)	
				CO chemisorption[c]	TEM[d]
Ru/Mo_2TiC_2	Mo_2TiC_2	2.1	90	1.4	1.2
Ru-NP/Mo_2TiC_2	Mo_2TiC_2	7.8	55	2.3	2.4

[a]*Determined by ICP-MS.* [b]*Determined from CO temperature programmed desorption (CO-TPD). Calculated based on the adsorption of one CO atom per exposed Ru atom.* [c]*Ru particle size was estimated from dispersion (particle size=1.29/D).* [d]*Ru particle size was obtained by measuring 150 Ru particles in TEM images.*

Table S3. Thermocatalytic performance of different catalysts

Catalyst	Temperature (K)	CO selectivity (%)	Conversion rate (mol/g_{Ru}/h)	CO_2 conversion degree
Ru/Mo_2TiC_2	473	98.1	0.04	1.2%
	573	98.3	0.16	2.0%
	673	99.4	2.22	4.1%
	773	99.9	7.84	7.7%
Ru-NP/Mo_2TiC_2	473	93.1	0.01	<1%
	573	99.2	0.12	1.1%
	673	99.4	0.63	2.9%
	773	99.7	2.20	6.4%
Ru/SiO_2	473	81.2	0.04	1.4%
	573	89.3	0.48	2.7%
	673	91.7	2.80	5.2%
	773	99.1	7.78	8.1%

Reaction conditions: Atmospheric pressure, 200-500°C, weight hourly space velocity (WHSV) = 20000 mL h^{-1} g_{cat}^{-1}, $H_2/CO_2/N_2$ = 25/25/50.

Table S4. Photothermal catalytic performance of different catalysts

Catalyst	Light intensity (W/cm^2)	CO selectivity (%)	Conversion rate ($mmol/g_{Ru}/h$)	CO_2 conversion degree (%)
Ru/Mo_2TiC_2	3.0	97.1	222	<1%
	3.2	99.1	311	<1%
	3.4	99.4	440	1.1%
	3.6	99.6	511	1.7%
	3.8	99.7	678	2.0%
$Ru-NP/Mo_2TiC_2$	3.0	97.1	166	<1%
	3.2	98.8	197	<1%
	3.4	99.2	254	<1%
	3.6	99.3	313	1.2%
	3.8	99.4	360	1.4%
Ru/SiO_2	3.0	/	/	/
	3.2	/	/	/
	3.4	81.1	1	/
	3.6	81.2	8	/
	3.8	84.8	8	/

Reaction conditions: Atmospheric pressure; Light intensity: 3.0-3.8 W/cm^2, weight hourly space velocity (WHSV) = 20000 mL h^{-1} g_{cat}^{-1}, $H_2/CO_2/N_2$ = 25/25/50.

Table S5. Performance of various Ru-based catalysts in photothermal CO_2 hydrogenation

Catalyst	Metal wt %	Reaction conditions[a]	R_{CO2} (mol/h/g_{cat})	H_2:CO_2 (Feed gas composition)	Space velocity (ml/g_{cat}/h)	CO_2 conversion (%)	Selectivity	
							CH_4 (%)	CO (%)
Ru/Al_2O_3[1]	2.4	300 W Xe light (batch reactor, no external heating)	0.44	~4.1:1	/	95	99	1
Ru/TiO_2[1]	2.5	300 W Xe light (batch reactor, no external heating)	5.3×10^{-9}	~4.1:1	/	/	100	0
Ru@FL-LDHs[2]	2.5	300 W Xe light (1.0 W/cm^2, flow reactor, 150℃)	0.27	4:1	10200	96	100	0
Ru/SiNW[3]	1.2	300 W Xe light (0.32 W/cm^2, batch reactor, 150℃)	1.1×10^{-4}	4:1	/	/	100	0
ncRuO_2/i-Si-o[4]	/	300 W Xe light (2.2 W/cm^2, batch reactor, no external heating)	4.4×10^{-3}	4:1	/	/	100	0
Ru/i-Si-o[5]	/	300 W Xe light (2.5 W/cm^2, batch reactor, 150℃)	2.8×10^{-3}	4:1	/	/	100	0
Ru/TiO_2[6]	1.0	500 W solar simulator (0.1 W/cm^2, flow reactor, 300℃)	6.9×10^{-2}	3:1	20000	32	100	0
Ru@$Ni_2V_2O_7$[7]	0.35	300 W Xe light (2.0 W/cm^2, batch reactor, no external heating)	0.11	~4.1:1	/	94	99	1
RuO_2/$SrTiO_3$[8]	0.40	300 W Xe light (0.1 W/cm^2, batch reactor, 150℃)	1.5×10^{-3}	4:1	/	90	99	1
**Ru/Mo_2TiC_2[This work]**	**2.1**	**300 W Xe light (3.8 W/cm^2, batch reactor, no external heating)**	**0.10**	**1:1**	**/**	**5**	**0**	**100**

[a]Reaction conditions consist of light source, operation mode and reaction temperature.

Table S6. Performance of various catalysts in photothermal RWGS.

Catalyst	Metal wt %	Reaction conditions[a]	$H_2:CO_2$	CO rate [mol/h/g]	Space velocity (ml/g_{cat}/h)	CO_2 conversion (%)
Fe/Al_2O_3[1]	2.4	300 W Xe light (batch reactor, no external heating)	4:1	1.9×10^{-2}	/	7.2
$In_2O_{3-x}(OH)_y$[9]	/	300 W Xe light (0.1 W/cm^2, flow reactor, no external heating)	1:1	1.5×10^{-4}	15000-45000	0.1
$In_2O_{3-x}(OH)_y$/SiNW[10]	/	300 W Xe light (2.0 W/cm^2, batch reactor, no external heating)	1:1	2.2×10^{-5}	/	0.1
Fe@C[11]	61.9	300 W Xe light (batch reactor, no external heating)	1:1	1.8×10^{-4}	/	4.5
$Ni_{12}P_5/SiO_2$[12]	/	300 W Xe light (2.3 W/cm^2, batch reactor, no external heating)	1:5	0.96	/	1.5
$Ni_{12}P_5@SiO_2$[13]	/	300 W Xe light (4.0 W/cm^2, batch reactor, no external heating)	1:1	0.43	120000	4.3
$Pd@Nb_2O_5$[14]	3.9	300 W Xe light (2.5 W/cm^2, batch reactor, no external heating)	1:1	4.9×10^{-3}	/	1.1
$In_2O_{3-x}(OH)_y$ nanorods[15]	/	1000 W Hortilux Blue metal halide bulb (0.8 W/cm^2, batch reactor, no external heating)	1:1	1.2×10^{-6}	/	0.5
Bi_2O_{3-x}[16]	/	420 nm LED (0.12 W/cm^2, batch reactor, no external heating)	1:2	1.6×10^{-5}	/	1.6
Co@CoN&C[17]	1.0	300 W Xe light (batch reactor, no external heating)	1:1	0.07	/	/
**Ru/Mo_2TiC_2[This work]**	**2.1**	**300 W Xe light (3.8 W/cm^2. batch reactor, no external heating)**	**1:1**	**4.0**	/	**2**
**Ru/Mo_2TiC_2[This work]**	**2.1**	**Natural sunlight (3.7 W/cm^2, batch reactor, no external heating)**	**1:1**	**3.9**	/	**1.8**

[a]Reaction conditions consist of light source, operation mode and reaction temperature.

Reference

1. Meng, X.; Wang, T.; Liu, L.; Ouyang, S.; Li, P.; Hu, H.; Kako, T.; Iwai, H.; Tanaka, A.; Ye, J., Photothermal conversion of CO_2 into CH_4 with H_2 over group VIII nanocatalysts: an alternative approach for solar fuel production. *Angew. Chem. Int. Ed*. **2014**, *53* (43), 11478-11482.

2. Ren, J.; Ouyang, S.; Xu, H.; Meng, X.; Wang, T.; Wang, D.; Ye, J., Targeting activation of CO_2 and H_2 over Ru-loaded ultrathin layered double hydroxides to achieve efficient photothermal CO_2 methanation in flow-type system. *Adv. Energy Mater*. **2017**, *7* (5), 1601657.

3. O'Brien, P. G.; Sandhel, A.; Wood, T. E.; Jelle, A. A.; Hoch, L. B.; Perovic, D. D.; Mims, C. A.; Ozin, G. A., Photomethanation of gaseous CO_2 over Ru/Silicon nanowire catalysts with visible and near-infrared photons. *Adv. Sci*. **2014**, *1* (1), 1400001.

4. Jelle, A. A.; Ghuman, K. K.; O'Brien, P. G.; Hmadeh, M.; Sandhel, A.; Perovic, D. D.; Singh, C. V.; Mims, C. A.; Ozin, G. A., Highly efficient ambient temperature CO_2 photomethanation catalyzed by nanostructured RuO_2 on silicon photonic crystal support. *Adv. Energy Mater*. **2018**, *8* (9), 1702277.

5. O’Brien, P. G.; Ghuman, K. K.; Jelle, A. A.; Sandhel, A.; Wood, T. E.; Loh, J. Y. Y.; Jia, J.; Perovic, D.; Singh, C. V.; Kherani, N. P.; Mims, C. A.; Ozin, G. A., Enhanced photothermal reduction of gaseous CO_2 over silicon photonic crystal supported ruthenium at ambient temperature. *Energy Environ. Sci*. **2018**, *11* (12), 3443-3451.

6. Wang, C.; Fang, S.; Xie, S.; Zheng, Y.; Hu, Y. H., Thermo-photo catalytic CO_2 hydrogenation over Ru/TiO_2. *J. Mater. Chem. A*. **2020**, *8* (15), 7390-7394.

7. Chen, Y.; Zhang, Y.; Fan, G.; Song, L.; Jia, G.; Huang, H.; Ouyang, S.; Ye, J.; Li, Z.; Zou, Z., Cooperative catalysis coupling photo-/photothermal effect to drive Sabatier reaction with unprecedented conversion and selectivity. *Joule* **2021**, *5* (12), 3235-3251.

8. Mateo, D.; Albero, J.; García, H. Titanium-perovskite-supported RuO_2 nanoparticles for photocatalytic CO_2 methanation. *Joule* 2019, *3* (8), 1949-1962.

9. Ghuman, K. K.; Wood, T. E.; Hoch, L. B.; Mims, C. A.; Ozin, G. A.; Singh, C. V., Illuminating CO_2 reduction on frustrated Lewis pair surfaces: investigating the role of surface hydroxides and oxygen vacancies on nanocrystalline $In_2O_{3-x}(OH)_y$. *Phys. Chem. Chem. Phys*. **2015**, *17* (22), 14623-14635.

10. Hoch, L. B.; O'Brien, P. G.; Jelle, A.; Sandhel, A.; Perovic, D. D.; Mims, C. A.; Ozin, G. A., Nanostructured indium oxide coated silicon nanowire arrays: a hybrid photothermal/photochemical approach to solar fuels. *ACS Nano* **2016**, *10* (9), 9017-9025.

11. Zhang, H.; Wang, T.; Wang, J.; Liu, H.; Dao, T. D.; Li, M.; Liu, G.; Meng, X.; Chang, K.; Shi, L.; Nagao, T.; Ye, J., Surface-plasmon-enhanced photodriven CO_2 reduction catalyzed by metal–organic-framework-derived iron nanoparticles encapsulated by ultrathin carbon layers. *Adv. Mater*. **2016**, *28* (19), 3703-3710.

12. Xu, Y.-F.; Duchesne, P. N.; Wang, L.; Tavasoli, A.; Jelle, A. A.; Xia, M.; Liao, J.-F.; Kuang, D.-B.; Ozin, G. A., High-performance light-driven heterogeneous CO_2 catalysis with near-unity selectivity on metal phosphides. *Nat. Commun*. **2020**, *11* (1), 5149-5156.

13. Lou, D.; Zhu, Z.; Xu, Y.-F.; Li, C.; Feng, K.; Zhang, D.; Lv, K.; Wu, Z.; Zhang, C.; Ozin, G. A.; He, L.; Zhang, X., A core-shell catalyst design boosts the performance of photothermal reverse water gas shift catalysis. *Sci. China Mater*. **2021**, *64* (9), 2212–2220.

14. Jia, J.; O'Brien, P. G.; He, L.; Qiao, Q.; Fei, T.; Reyes, L. M.; Burrow, T. E.; Dong, Y.; Liao, K.; Varela, M.; Pennycook, S. J.; Hmadeh, M.; Helmy, A. S.; Kherani, N. P.; Perovic, D. D.; Ozin, G. A., Visible and near-infrared photothermal catalyzed hydrogenation of gaseous CO_2 over nanostructured Pd@Nb_2O_5. *Adv. Sci.* **2016**, *3* (10), 1600189.

15. He, L.; Wood, T. E.; Wu, B.; Dong, Y.; Hoch, L. B.; Reyes, L. M.; Wang, D.; Kübel, C.; Qian, C.; Jia, J.; Liao, K.; O'Brien, P. G.; Sandhel, A.; Loh, J. Y. Y.; Szymanski, P.; Kherani, N. P.; Sum, T. C.; Mims, C. A.; Ozin, G. A., Spatial separation of charge carriers in $In_2O_{3-x}(OH)_y$ nanocrystal superstructures for enhanced gas-phase photocatalytic activity. *ACS Nano* **2016**, *10* (5), 5578-5586.

16. Li, Y.; Wen, M.; Wang, Y.; Tian, G.; Wang, C.; Zhao, J., Plasmonic hot electrons from oxygen vacancies for infrared light-driven catalytic CO_2 reduction on Bi_2O_{3-x}. *Angew. Chem. Int. Ed.* **2021**, *60* (2), 910-916.

17. Ning, S.; Xu, H.; Qi, Y.; Song, L.; Zhang, Q.; Ouyang, S.; Ye, J., Microstructure induced thermodynamic and kinetic modulation to enhance CO_2 photothermal reduction: A case of atomic-scale dispersed Co–N species anchored Co@C hybrid. *ACS Catal.* **2020**, *10* (8), 4726-4736.
